# Withdrawal of heart failure therapy after atrial fibrillation rhythm control with ejection fraction normalization: the WITHDRAW-AF trial

**DOI:** 10.1093/eurheartj/ehaf563

**Published:** 2025-08-12

**Authors:** Louise Segan, Peter M Kistler, Shane Nanayakkara, Andrew Taylor, James Hare, Benedict Costello, David Chieng, Rose Crowley, Jeremy William, Hariharan Sugumar, Kenneth Cho, Aleksandr Voskoboinik, Liang-Han Ling, Ziporah Nderitu, Sonia Azzopardi, Annie Curtin, Manuja Premaratne, Alex J McLellan, Justin Mariani, Joseph B Morton, Geoffrey Lee, Stephen Joseph, Christopher Reid, David M Kaye, Jonathan M Kalman, Sandeep Prabhu

**Affiliations:** Department of Cardiometabolics, The Baker Heart and Diabetes Institute, 99 Commercial Rd, Melbourne, VIC 3004, Australia; Heart Centre at the Alfred Hospital, 55 Commercial Road, Melbourne 3004, Australia; Faculty of Medicine, Dentistry and Health Sciences, University of Melbourne, 138-146 Cardigan St, Carlton, VIC 3053, Australia; Department of Medicine, Monash University, Wellington Rd, Clayton, VIC 3800, Australia; Department of Cardiology, Cabrini Hospital, 181/183 Wattletree Rd, Malvern, VIC 3144, Australia; Department of Cardiometabolics, The Baker Heart and Diabetes Institute, 99 Commercial Rd, Melbourne, VIC 3004, Australia; Heart Centre at the Alfred Hospital, 55 Commercial Road, Melbourne 3004, Australia; Faculty of Medicine, Dentistry and Health Sciences, University of Melbourne, 138-146 Cardigan St, Carlton, VIC 3053, Australia; Department of Medicine, Monash University, Wellington Rd, Clayton, VIC 3800, Australia; Department of Cardiology, Cabrini Hospital, 181/183 Wattletree Rd, Malvern, VIC 3144, Australia; Department of Cardiology, Melbourne Private Hospital, 1F Royal Parade, Parkville, VIC 3050, Australia; Department of Cardiometabolics, The Baker Heart and Diabetes Institute, 99 Commercial Rd, Melbourne, VIC 3004, Australia; Heart Centre at the Alfred Hospital, 55 Commercial Road, Melbourne 3004, Australia; Department of Medicine, Monash University, Wellington Rd, Clayton, VIC 3800, Australia; Department of Cardiology, Cabrini Hospital, 181/183 Wattletree Rd, Malvern, VIC 3144, Australia; Department of Cardiometabolics, The Baker Heart and Diabetes Institute, 99 Commercial Rd, Melbourne, VIC 3004, Australia; Heart Centre at the Alfred Hospital, 55 Commercial Road, Melbourne 3004, Australia; Faculty of Medicine, Dentistry and Health Sciences, University of Melbourne, 138-146 Cardigan St, Carlton, VIC 3053, Australia; Department of Cardiology, The Royal Melbourne Hospital, 300 Grattan St, Parkville, VIC 3050, Australia; Department of Cardiometabolics, The Baker Heart and Diabetes Institute, 99 Commercial Rd, Melbourne, VIC 3004, Australia; Heart Centre at the Alfred Hospital, 55 Commercial Road, Melbourne 3004, Australia; Department of Cardiometabolics, The Baker Heart and Diabetes Institute, 99 Commercial Rd, Melbourne, VIC 3004, Australia; Department of Cardiology, Western Health, 176 Furlong Rd, St Albans, VIC 3021, Australia; Department of Cardiometabolics, The Baker Heart and Diabetes Institute, 99 Commercial Rd, Melbourne, VIC 3004, Australia; Heart Centre at the Alfred Hospital, 55 Commercial Road, Melbourne 3004, Australia; Faculty of Medicine, Dentistry and Health Sciences, University of Melbourne, 138-146 Cardigan St, Carlton, VIC 3053, Australia; Department of Medicine, Monash University, Wellington Rd, Clayton, VIC 3800, Australia; Department of Cardiology, Cabrini Hospital, 181/183 Wattletree Rd, Malvern, VIC 3144, Australia; Department of Cardiometabolics, The Baker Heart and Diabetes Institute, 99 Commercial Rd, Melbourne, VIC 3004, Australia; Heart Centre at the Alfred Hospital, 55 Commercial Road, Melbourne 3004, Australia; Faculty of Medicine, Dentistry and Health Sciences, University of Melbourne, 138-146 Cardigan St, Carlton, VIC 3053, Australia; Department of Medicine, Monash University, Wellington Rd, Clayton, VIC 3800, Australia; Department of Cardiology, Cabrini Hospital, 181/183 Wattletree Rd, Malvern, VIC 3144, Australia; Department of Cardiometabolics, The Baker Heart and Diabetes Institute, 99 Commercial Rd, Melbourne, VIC 3004, Australia; Heart Centre at the Alfred Hospital, 55 Commercial Road, Melbourne 3004, Australia; Faculty of Medicine, Dentistry and Health Sciences, University of Melbourne, 138-146 Cardigan St, Carlton, VIC 3053, Australia; Department of Medicine, Monash University, Wellington Rd, Clayton, VIC 3800, Australia; Department of Cardiology, Cabrini Hospital, 181/183 Wattletree Rd, Malvern, VIC 3144, Australia; Department of Cardiometabolics, The Baker Heart and Diabetes Institute, 99 Commercial Rd, Melbourne, VIC 3004, Australia; Heart Centre at the Alfred Hospital, 55 Commercial Road, Melbourne 3004, Australia; Faculty of Medicine, Dentistry and Health Sciences, University of Melbourne, 138-146 Cardigan St, Carlton, VIC 3053, Australia; Department of Medicine, Monash University, Wellington Rd, Clayton, VIC 3800, Australia; Department of Cardiology, Cabrini Hospital, 181/183 Wattletree Rd, Malvern, VIC 3144, Australia; Department of Cardiometabolics, The Baker Heart and Diabetes Institute, 99 Commercial Rd, Melbourne, VIC 3004, Australia; Heart Centre at the Alfred Hospital, 55 Commercial Road, Melbourne 3004, Australia; Faculty of Medicine, Dentistry and Health Sciences, University of Melbourne, 138-146 Cardigan St, Carlton, VIC 3053, Australia; Department of Cardiometabolics, The Baker Heart and Diabetes Institute, 99 Commercial Rd, Melbourne, VIC 3004, Australia; Heart Centre at the Alfred Hospital, 55 Commercial Road, Melbourne 3004, Australia; Faculty of Medicine, Dentistry and Health Sciences, University of Melbourne, 138-146 Cardigan St, Carlton, VIC 3053, Australia; Department of Medicine, Monash University, Wellington Rd, Clayton, VIC 3800, Australia; Department of Cardiology, Cabrini Hospital, 181/183 Wattletree Rd, Malvern, VIC 3144, Australia; Department of Cardiometabolics, The Baker Heart and Diabetes Institute, 99 Commercial Rd, Melbourne, VIC 3004, Australia; Heart Centre at the Alfred Hospital, 55 Commercial Road, Melbourne 3004, Australia; Faculty of Medicine, Dentistry and Health Sciences, University of Melbourne, 138-146 Cardigan St, Carlton, VIC 3053, Australia; Department of Cardiology, St Vincent’s Private Hospital Fitzroy, 41 Victoria Parade, Fitzroy, VIC 3065, Australia; Department of Cardiometabolics, The Baker Heart and Diabetes Institute, 99 Commercial Rd, Melbourne, VIC 3004, Australia; Heart Centre at the Alfred Hospital, 55 Commercial Road, Melbourne 3004, Australia; Department of Cardiometabolics, The Baker Heart and Diabetes Institute, 99 Commercial Rd, Melbourne, VIC 3004, Australia; Heart Centre at the Alfred Hospital, 55 Commercial Road, Melbourne 3004, Australia; Department of Cardiometabolics, The Baker Heart and Diabetes Institute, 99 Commercial Rd, Melbourne, VIC 3004, Australia; Heart Centre at the Alfred Hospital, 55 Commercial Road, Melbourne 3004, Australia; Department of Medicine, Monash University, Wellington Rd, Clayton, VIC 3800, Australia; Faculty of Medicine, Dentistry and Health Sciences, University of Melbourne, 138-146 Cardigan St, Carlton, VIC 3053, Australia; Department of Cardiology, Melbourne Private Hospital, 1F Royal Parade, Parkville, VIC 3050, Australia; Department of Cardiology, The Royal Melbourne Hospital, 300 Grattan St, Parkville, VIC 3050, Australia; Department of Cardiology, St Vincent’s Private Hospital Fitzroy, 41 Victoria Parade, Fitzroy, VIC 3065, Australia; Department of Cardiometabolics, The Baker Heart and Diabetes Institute, 99 Commercial Rd, Melbourne, VIC 3004, Australia; Heart Centre at the Alfred Hospital, 55 Commercial Road, Melbourne 3004, Australia; Department of Cardiology, St Vincent’s Private Hospital Fitzroy, 41 Victoria Parade, Fitzroy, VIC 3065, Australia; Faculty of Medicine, Dentistry and Health Sciences, University of Melbourne, 138-146 Cardigan St, Carlton, VIC 3053, Australia; Department of Cardiology, Melbourne Private Hospital, 1F Royal Parade, Parkville, VIC 3050, Australia; Department of Cardiology, The Royal Melbourne Hospital, 300 Grattan St, Parkville, VIC 3050, Australia; Faculty of Medicine, Dentistry and Health Sciences, University of Melbourne, 138-146 Cardigan St, Carlton, VIC 3053, Australia; Department of Cardiology, Melbourne Private Hospital, 1F Royal Parade, Parkville, VIC 3050, Australia; Department of Cardiology, The Royal Melbourne Hospital, 300 Grattan St, Parkville, VIC 3050, Australia; Department of Cardiology, Epworth Hospital Richmond, 89 Bridge Rd, Richmond, VIC 3121, Australia; Department of Cardiology, Cabrini Hospital, 181/183 Wattletree Rd, Malvern, VIC 3144, Australia; Department of Cardiology, Western Health, 176 Furlong Rd, St Albans, VIC 3021, Australia; Faculty of Health Sciences, Curtin University, Kent St, Bentley, WA 6102, Australia; Department of Cardiometabolics, The Baker Heart and Diabetes Institute, 99 Commercial Rd, Melbourne, VIC 3004, Australia; Heart Centre at the Alfred Hospital, 55 Commercial Road, Melbourne 3004, Australia; Department of Medicine, Monash University, Wellington Rd, Clayton, VIC 3800, Australia; Faculty of Medicine, Dentistry and Health Sciences, University of Melbourne, 138-146 Cardigan St, Carlton, VIC 3053, Australia; Department of Cardiology, Melbourne Private Hospital, 1F Royal Parade, Parkville, VIC 3050, Australia; Department of Cardiology, The Royal Melbourne Hospital, 300 Grattan St, Parkville, VIC 3050, Australia; Department of Cardiometabolics, The Baker Heart and Diabetes Institute, 99 Commercial Rd, Melbourne, VIC 3004, Australia; Heart Centre at the Alfred Hospital, 55 Commercial Road, Melbourne 3004, Australia; Faculty of Medicine, Dentistry and Health Sciences, University of Melbourne, 138-146 Cardigan St, Carlton, VIC 3053, Australia; Department of Medicine, Monash University, Wellington Rd, Clayton, VIC 3800, Australia; Department of Cardiology, Epworth Hospital Richmond, 89 Bridge Rd, Richmond, VIC 3121, Australia; Department of Cardiology, Mulgrave Private Hospital, 48 Blanton Dr, Mulgrave, VIC 3170, Australia

**Keywords:** Atrial fibrillation, Normalized left ventricular ejection fraction, Catheter ablation, Clinical trial, Arrhythmia-induced cardiomyopathy, AF-mediated cardiomyopathy, Heart failure therapy

## Abstract

**Background and Aims:**

Atrial fibrillation-mediated cardiomyopathy (AFCM) represents an important reversible cause of left ventricular systolic dysfunction. Current clinical practice is indefinite heart failure (HF) pharmacotherapy despite left ventricular ejection fraction (LVEF) normalization. However, whether this is necessary to maintain normal LVEF, in addition to rhythm control, is uncertain.

**Methods:**

This multi-centre, randomized trial conducted between 2021 and 2024 examined the impact of staged withdrawal of HF therapy following AF rhythm control and LVEF normalization in AFCM. Participants were randomized (1:1) to early withdrawal (Group A) or continued therapy for 6 months followed by delayed withdrawal (Group B), in a crossover design. The primary endpoint was the randomized comparison of cardiac magnetic resonance (CMR) LVEF maintenance ≥50% at 6 months, during which time Group A had withdrawn therapy and Group B remained on treatment. Secondary outcomes included cardiac remodelling, functional status, biomarkers, quality of life, and arrhythmia recurrence on vs off HF therapy. The total follow-up duration was 12 months.

**Results:**

Between July 2021 and May 2024, 60 patients were enrolled (age 60 [55–65] years, previous persistent AF <1 year and maintaining sinus rhythm for minimum 6 months following AF rhythm control [catheter ablation in 97%]). All participants completed treatment withdrawal and 12-month follow-up. In the initial randomized comparison, LVEF was maintained ≥50% at 6 months in 90% of participants undergoing HF therapy withdrawal (Group A), compared with 100% who continued medical therapy (Group B) (odds ratio [OR] 1.18, 95% confidence interval [CI] 0.27–2.82, *P* = .47). CMR LVEF was similar between randomization groups at the end of the randomization phase (Group A: LVEF 58% [95% CI 54–60] vs Group B: LVEF 59% [95% CI 55–64], *P* = .236) and across study time points (mixed effects *P* = .37). Transthoracic echocardiography characteristics, N-terminal pro-B-type natriuretic peptide, functional status, quality of life and AF burden were similar on vs off HF therapy in the overall population.

**Conclusions:**

Withdrawal of HF therapy following AF rhythm control for prior AFCM and recovered LVEF was not associated with a decline in LVEF for most patients in the following 6 months.

## Structured Graphical Abstract

**Structured Graphical Abstract:**
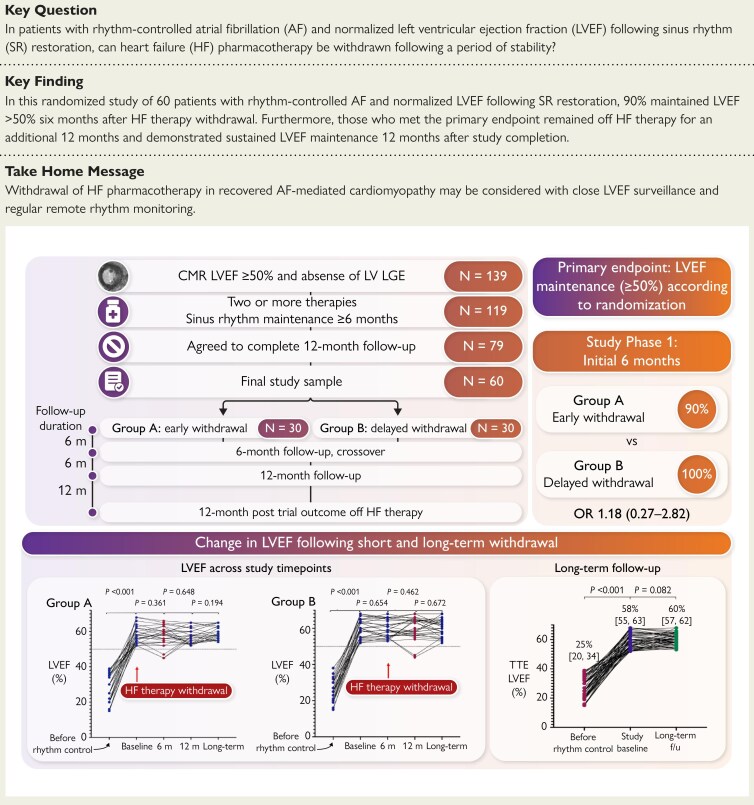
In individuals with presumed atrial fibrillation-mediated cardiomyopathy and normalized left ventricular ejection fraction (LVEF) following AF rhythm control, staged withdrawal of heart failure (HF) therapy was feasible, with 90% maintaining LVEF ≥50% and stable left ventricular systolic function at long-term follow-up (f/u). Those who relapsed showed prompt recovery after therapy re-initiation, supporting a cautious, individualized withdrawal approach in selected patients. CMR, cardiac magnetic resonance; LGE, late gadolinium enhancement; LV, left ventricular; OR, odds ratio; TTE, transthoracic echocardiography.

**See the editorial comment for this article ‘Managing atrial fibrillation-mediated cardiomyopathy: the causality-consequence conundrum', by T. Baykaner and J. Butler, https://doi.org/10.1093/eurheartj/ehaf764.**

## Introduction

Heart failure (HF) represents a sizable global health burden (affecting over 55 million people worldwide) and is responsible for substantial healthcare resource utilization globally.^1^ Arrhythmia-induced cardiomyopathy (AiCM) is a common reversible cause of left ventricular (LV) systolic dysfunction (LVSD); atrial fibrillation (AF) accounts for the vast majority of AiCM (92%; termed AF-mediated cardiomyopathy (AFCM)).^2^ AFCM is associated with significant improvements and often normalization in LV systolic function following sinus rhythm (SR) restoration.^3–5^ Current guidelines recommend indefinite HF pharmacotherapy following normalization of LV ejection fraction (LVEF) in dilated cardiomyopathy (DCM), irrespective of aetiology, to maintain LVEF.^6^ However, whether indefinite HF pharmacotherapy remains necessary, over and above rhythm control, to maintain LVEF in AFCM, is uncertain.^7^

This study sought to evaluate the effect of short-term withdrawal of guideline-directed medical therapy (GDMT) for HF in patients with AFCM with established rhythm control following LVEF normalization.

## Methods

### Study design and patient population

**Figure 1:**
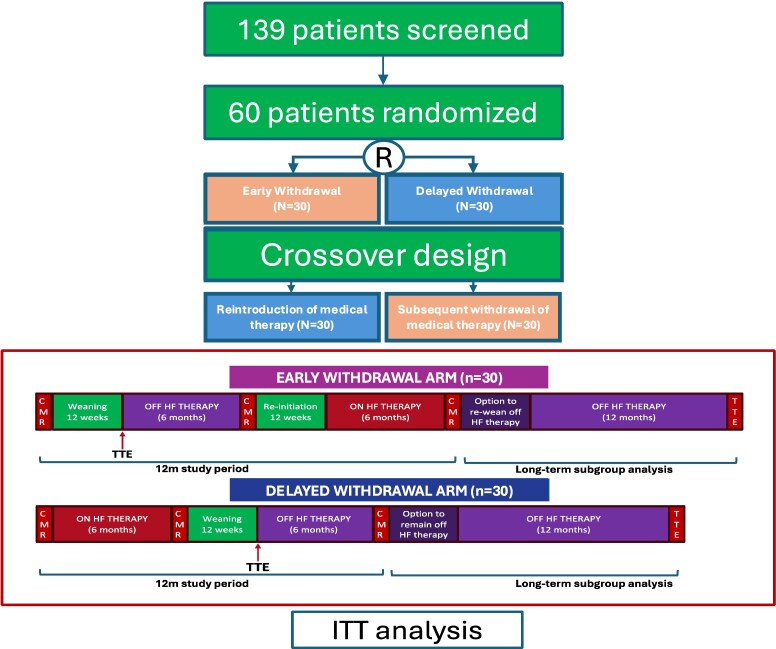
Study protocol. Participants were randomized to either initial medication withdrawal (top) or continued medical therapy (bottom) for 6 months, followed by double crossover to the alternate treatment arm. Participants who demonstrated maintenance of left ventricular ejection fraction (LVEF) ≥50% following medication withdrawal were offered the option to remain off heart failure (HF) therapy for a further 12 months following study completion in a pre-specified long-term follow-up sub-analysis with transthoracic echocardiography performed 12 months following study completion to monitor long-term LVEF off HF pharmacotherapy

This was a multi-centre open-label randomized crossover trial with blinded endpoint evaluation conducted across four Australian centres between July 2021 and May 2024 (*Figure 1*). Patients eligible for enrolment included individuals with rhythm-controlled AF and normalized LVEF following SR restoration receiving ≥2 HF agents and without cardiac magnetic resonance (CMR)-detected ventricular late gadolinium enhancement (LGE). All participants provided written informed consent and the potential risks of HF and arrhythmia recurrence was discussed. Individuals were enrolled if all inclusion criteria and no exclusion criteria were met (see Supplementary data online, Table  *S1*). The trial was granted ethical approval from the Alfred Hospital Human Ethics and Research Committee and at participating institutions. Annual progress reports and quarterly safety monitoring reports were submitted. The study design was discussed with a HF advisory group from the trial site.

### Study inclusion

Inclusion criteria included: (i) a prior diagnosis of LVSD (LVEF <40%) at LVSD diagnosis during AF; (ii) absence of current HF symptoms; (iii) clinically euvolemic state; (iv) current treatment with two or more HF therapies (loop diuretic, mineralocorticoid receptor antagonist [MRA], angiotensin-converting enzyme inhibitor [ACEi], angiotensin receptor blocker [ARB], angiotensin receptor–neprilysin inhibitor [ARNi] or beta-blockade); (v) current LVEF ≥50% on screening visit CMR; (vi) no evidence of CMR-detected LV myocardial fibrosis on delayed enhancement imaging; and (vii) plasma N-terminal pro-B-type natriuretic peptide (NT-proBNP) concentration <250 ng/l. Normalized LVEF was defined as improvement from prior LVEF <40%–≥50% at enrolment and supported by the following: (i) NT-proBNP <250 ng/L; (ii) New York Heart Association (NYHA) Class I symptoms; and (iii) maintenance of SR over the preceding 6 months, according to prior publications.^8^ The NT-proBNP threshold was applied to exclude individuals with persistently elevated natriuretic peptide levels despite LVEF normalization, thereby identifying a cohort with biochemical evidence of cardiac normalization and a lower likelihood of sub-clinical HF.

### Study exclusion

Exclusion criteria comprised: (i) inability to maintain SR; (ii) ventricular arrhythmias in whom beta-blockade cessation was deemed inappropriate; (iii) known alternate cardiomyopathy aetiology; (iv) visually apparent LV LGE on screening CMR; (v) significant renal impairment (estimated glomerular filtration rate <30 ml/min/1.73 m^2^) in whom administration of gadolinium contrast may be contraindicated; (vi) significant valvular disease (moderate or greater severity); (vii) an indication for ACEi/ARB other than LVSD (such as Type 2 diabetes mellitus or uncontrolled hypertension requiring >1 agent in whom cessation may worsen systemic hypertension); and (viii) age younger than 18 years (see Supplementary data online, Table  *S1*).

### Randomization

Participants were randomized (1:1) to undergo either *early withdrawal* (Group A) or *delayed withdrawal* (Group B) of HF therapy. These labels refer to the study time point at which medication withdrawal occurred—immediately following enrolment (Group A) or after a 6-month continuation phase (Group B)—and do not reflect differences in the withdrawal strategy or protocol itself, which was identical across both groups. A period of 6 months off HF therapy was pre-specified as a safety measure given the findings from TRED-HF, which reported a relapse rate of 44% within 6 months among a diverse DCM population.^8^ Randomization was computer generated. The initial randomized phase took place over 6 months after a 12-week run in phase of medication weaning according to the study protocol (see Supplementary data online, Figure  *S2* and Appendix  *S1*). At the 6-month mark, participants crossed over to the alternate treatment arm (see Supplementary data online, Figure  *S2* and Appendix  *S2*). All participants underwent HF therapy withdrawal at some point during the 12-month study period (see Supplementary data online, Figure  *S2*).

The crossover design was selected to ensure: (i) similar follow-up between groups; (ii) each subject served as their own control to mitigate confounding and for secondary analyses; and (iii) to examine whether the timing of treatment withdrawal influenced the primary outcome. CMR and transthoracic echocardiography (TTE) imaging analysis was performed in a core lab blinded to treatment assignment and study time points. Outcome assessors were blinded to allocation and treatment stage; study investigators and patients were aware of allocation.

### Intervention: treatment withdrawal

Supervised withdrawal comprised a stepwise reduction in HF pharmacotherapy every 2 weeks up to a maximum of 12 weeks, adapted from the TRED-HF protocol.^8^ However, unlike the original TRED-HF medication weaning protocol, in the present study, beta-blockers were weaned first to allow earlier detection of atrial arrhythmias in this AFCM population, where beta-blockers might otherwise suppress early signs of relapse due to arrhythmia recurrence. This modification enabled timely reinstatement of rhythm control if needed. A detailed description is provided in Supplementary data online, Figure  *S2* and Appendix  *S1*. In summary, medication weaning commenced with: (i) reduction and/or cessation of diuretic agents; (ii) reduction and/or cessation of beta-blockers (bisoprolol, metoprolol XL, nebivolol, carvedilol); (iii) reduction and/or cessation of MRA, and (iv) lastly, reduction and/or cessation of ACEi/ARB/ARNi. Participants underwent intensive surveillance with daily blood pressure, heart rate and body weight measurements and twice daily ECG transmissions. Interval TTE was performed 4 weeks following complete medication cessation for patient safety. Following the completion of medication withdrawal, a further 2-week washout period was implemented before the 6-month ‘off therapy’ follow-up period commenced, to allow for pharmacological clearance of HF therapies. This was based on established pharmacokinetic principles, whereby 5–7 half-lives is considered sufficient for >99% drug elimination and exceeds the known elimination half-life for all agents withdrawn in this study.^9^

Patients who did not exhibit LVEF reduction at the interim TTE (4 weeks post-weaning) remained off HF therapy for a total of 6 months. Remote rhythm monitoring with single lead ECG or implanted cardiac device (where present) was undertaken in all participants and rhythm control was instituted for arrhythmia episodes where appropriate (anti-arrhythmic drug therapy, cardioversion and/or catheter ablation). Patients who developed hypertension without LVSD relapse were managed with calcium channel blockers (amlodipine). All patients had access to a trial doctor throughout the study.

### Outcomes

The primary endpoint was maintenance of CMR LVEF ≥50% at 6 months assessed by blinded endpoint evaluation and compared between randomized groups (Group A vs Group B). Secondary outcomes, including cardiac dimensions (LV and left atrial volume), LVEF, NT-proBNP, functional capacity, HF symptoms, resting heart rate, mean blood pressure, quality of life, arrhythmia recurrence (defined as AF/atrial tachycardia >30 s) and AF burden as well as safety (composite of major adverse cardiovascular events [MACE], HF hospitalization or cardiovascular mortality) were compared on vs off HF pharmacotherapy across the overall cohort using a within-subject design. For Group A (initial withdrawal), ‘on therapy’ values were derived from baseline (study enrolment) and ‘off therapy’ values from 6 months (following HF therapy withdrawal). For Group B (subsequent withdrawal at 6 months), ‘on therapy’ values were taken at 6 months and ‘off therapy’ values at 12 months (following crossover). This approach enabled each patient to serve as their own control for paired comparisons of physiologic and biochemical measures while on vs off HF therapy.

The methodology for AF burden quantification has been published previously^10^ and is outlined in Supplementary data online, Appendix  *S3*. AF burden was assessed using either an implanted cardiac device (where applicable) or twice-daily ECG transmissions using KardiaMobile^TM^ devices. AF burden was expressed as a percentage of time in AF over the follow-up period. A pre-specified analysis of LVEF reduction >5% and >10% post-short-term medication withdrawal was conducted, to assess for potential reductions in LVEF that did not meet the pre-specified threshold for the primary endpoint and which has been utilized as a threshold for significance in prior studies.^11^ The primary analysis was by intention to treat.

To assess the long-term durability of LVEF maintenance, participants who maintained LVEF ≥50% off HF therapy at the study conclusion were offered re-weaning or to remain off HF therapy for an additional 12 months, with follow-up TTE for longer-term LVEF surveillance.

### Statistical analysis

There are no randomized studies examining medication withdrawal in the AFCM population. Therefore, in order to estimate the required sample size, we performed a power calculation based on the results from AF patients in the TRED-HF study.^8^ The TRED-HF randomized study reported a relapse in LVSD in 44% among 51 patients with DCM following medication withdrawal. Within the AF sub-population, LVSD relapse occurred in 27.3%. On this basis and with a 0% event rate in the continuing medical therapy group (as demonstrated in TRED-HF), 60 patients (30 per treatment group) in the present study would be required to provide statistical power of 80%^12^ to detect a difference with the probability of a type one error being .05 and allowing for 10% loss to follow-up. This calculation was performed as a one-sided test.

Baseline characteristics were compared according to allocation. The primary outcome was analysed in the intention-to-treat population, defined as all participants who provided informed consent and were randomized according to the study protocol. Per protocol analysis yielded the same findings given there was no unplanned crossover.

The primary outcome (LVEF maintenance ≥50% at 6 months) was compared between randomized groups (Group A vs Group B) using logistic regression. Group assignment was the sole covariate in this mode, used to estimate the odds ratio (OR) and 95% confidence interval (CI) for the primary comparison.

Pre-specified analyses included the proportion with LVEF reduction >5% and >10% following medication withdrawal and subgroup analyses examining predictors of LVSD relapse: age (<65 vs ≥65 years), sex (male vs female), LVEF at the time of LVSD diagnosis (<30% vs ≥30%) enrolment LVEF (≤55% vs >55%), number of HF agents (≤2 vs >2) and maximal oxygen consumption (VO_2_max) (<20 vs ≥20 ml/kg/min). To adjust for multiple comparisons, a Bonferroni correction was applied. All secondary analyses were pre-specified (cardiac imaging characteristics, haemodynamics, functional status, biomarkers and quality of life) and were compared on vs off HF pharmacotherapy.

Multi-variable logistic regression was performed as a secondary exploratory analysis to identify baseline clinical, functional, and imaging characteristics associated with LVEF maintenance. Covariates were selected based on clinical relevance and included age, female sex, LVEF at the time of LVSD diagnosis, time to LVEF normalization and enrolment LVEF, NT-proBNP, VO_2_max, and left atrial volume index. Model goodness-of-fit was assessed using the Hosmer–Lemeshow test. Multi-collinearity was evaluated using variance inflation factors (VIFs) and covariates with VIF >5 were not included in the same model.

Continuous data are presented as means (±standard deviation) or median (inter-quartile range [IQR]) and compared using the Wilcoxon rank-sum and two-sample *t*-tests, as appropriate. Categorical variables were presented as counts and percentages and compared using the χ^2^ or Fisher’s exact test. The primary outcome utilized exact logistic regression, with measures of variance expressed utilizing Wilson’s score CIs given the modest sample size. A mixed-effects model was used to account for repeated LVEF measures between study groups and across time points. Secondary outcomes were compared on and off HF therapy using repeated measures ANOVA. Within-group comparisons of LVEF between time points (i.e. baseline vs 6 months, 6 months vs 12 months) were performed using the Wilcoxon signed-rank test. The Mann–Whitney *U* test was used for between-group comparisons at individual time points. All analyses were performed in the intention-to-treat population. A two-sided *P*-value of <.05 was considered to indicate significance for secondary analyses. Analyses were performed using R software (version 4.2.1, R Core Team).

## Results

### Study population

Between July 2021 and May 2024, 139 subjects were screened and 60 patients were deemed eligible and randomized 1:1 to either initial medication withdrawal (Group A, *n* = 30) or continued medical therapy (Group B, *n* = 30) (see Supplementary data online, Figure  *S1*). Baseline characteristics were similar between groups (*Table 1*). The majority (97%) underwent AF ablation (radiofrequency) prior to enrolment. All participants were in SR at enrolment, completed 12-month follow-up according to group assignment and had no missing data.

**Table 1:** Baseline characteristics according to randomization

	Randomization	
	Group A (early withdrawal)	Group B (delayed withdrawal)
	*N* = 30	*N* = 30
Baseline characteristics		
Sex		
Male, *n* (%)	27 (90)	22 (73)
Female, *n* (%)	3 (10)	8 (27)
Age, median (IQR)	61 [56, 64]	59 [55, 65]
BMI, kg/m^2^, median (IQR)	29 [26, 32]	29 [27, 32]
Hypertension, *n* (%)	2 (7)	1 (3)
Type 2 Diabetes, *n* (%)	2 (7)	1 (3)
Hyperlipidaemia, *n* (%)	10 (33)	7 (23)
Stroke, *n* (%)	1 (3)	1 (3)
OSA, *n* (%)	3 (10)	8 (27)
CKD, *n* (%)	1 (3)	2 (7)
Alcohol (>7 standard drinks/weeks), *n* (%)	4 (13)	3 (10)
Smoker, *n* (%)		
Never	18 (60)	24 (80)
Ex-smoker	10 (33)	6 (20)
Current smoker	2 (7)	0 (0)
LVEF at LVSD diagnosis, %	27 [20, 35]	25 [20, 32]
LVEDD at LVSD diagnosis, mm	58 [53, 60]	56 [52, 58]
Time since LVSD diagnosis, months, median (IQR)	19.6 [14.0, 32.1]	20.3 [15.5, 37.3]
Time since LV recovery, months, median (IQR)	14.1 [8.5, 17.8]	15.7 [8.0, 20.9]
Time from ablation to enrolment, median (IQR)	16.6 [8.3, 25.3]	16.7 [9.5, 21.0]
Prior ablation, *n* (%)	29 (97)	29 (97)
PVI only, *n* (%)	19 (66)	23 (79)
PVI + PWI, *n* (%)	10 (34)	6 (21)
Median no. HF agents, median (IQR)	2 [2, 3]	2 [2, 3]
Number of HF agents		
Two agents, *n* (%)	18 (60)	17 (57)
Three agents, *n* (%)	9 (30)	8 (27)
≥4 agents, *n* (%)	3 (10)	5 (16)
ARNi, *n* (%)	11 (37)	12 (40)
ACEi, *n* (%)	12 (40)	13 (43)
ARB, *n* (%)	7 (23)	5 (17)
MRA, *n* (%)	10 (33)	10 (33)
Furosemide, *n* (%)	2 (7)	5 (17)
SGLT2i, *n* (%)	4 (13)	2 (7)
Beta blocker, *n* (%)	30 (100)	30 (100)
Digoxin, *n* (%)	4 (13)	2 (7)
AAD, *n* (%)	3 (10)	5 (17)
Anti-coagulation, *n* (%)	27 (90)	29 (97)
ECG characteristics		
Baseline QRS width, ms	97 [91, 103]	94 [86, 101]
CMR characteristics		
LVEF, %, median (IQR)	58 [53, 61]	60 [55, 65]
CMR LVEDVi, ml/m^2^, median (IQR)	80 [73, 93]	81 [66, 86]
CMR LVESVi, ml/m^2^, median (IQR)	36 [29, 40]	30 [26, 34]
Native T1 time, ms, median (IQR)	1170 [1160, 1173]	1172 [1100, 1174]
TTE characteristics		
LVEDD, mm, median (IQR)	52 [48, 55]	52 [47, 55]
LVEF, %, median (IQR)	60 [55, 63]	60 [54, 62]
TTE LV GLS, %, median (IQR)	−18.0 [−17.0, −19.8]	−18.5 [−17.2, −20.1]
LVMI, g/m^2^, median (IQR)	80 [68, 98]	76 [69, 93]
TAPSE, mm, median (IQR)	22 [21, 23]	23 [21, 24]
LAVI, ml/m^2^, median (IQR)	36 [30, 45]	38 [32, 42]
LAA, cm^2^, median (IQR)	24 [20, 28]	24 [21, 26]
RAA, cm^2^, median (IQR)	20 [16, 21]	17 [15, 20]
Remote rhythm monitoring		
ICD, *n* (%)	2 (7)	1 (3)
PPM, *n* (%)	0 (0)	1 (3)
ILR, *n* (%)	1 (3)	1 (3)
Kardia device, *n* (%)	27 (90)	27 (90)

BMI, body mass index; OSA, obstructive sleep apnoea; CKD, chronic kidney disease (defined as eGFR <60 ml/min/1.73 m^2^); LVEF, left ventricular ejection fraction; LVSD, left ventricular systolic dysfunction; LVEDD, left ventricular end-diastolic diameter; IQR, inter-quartile range; PVI, pulmonary vein isolation; PWI, posterior wall isolation; HF, heart failure; ARNi, angiotensin receptor/neprilysin inhibitor; ACEi, angiotensin-converting enzyme inhibitor; ARB, angiotensin II receptor blocker; MRA, mineralocorticoid receptor antagonist; SGLT2i, sodium–glucose co-transporter-2 inhibitors; AAD, anti-arrhythmic drug; LVEDVi, left ventricular end-diastolic volume index; CMR, cardiac magnetic resonance imaging; LVESVi, left ventricular end-systolic volume index; TTE, transthoracic echocardiography; LV GLS, left ventricular global longitudinal strain; LVMI, left ventricular mass index; TAPSE, tricuspid annular plane systolic excursion; LAVI, left atrial volume index; LAA, left atrial area; RAA, right atrial area; ICD, implantable cardioverter-defibrillator; PPM, permanent pacemaker; ILR, implantable loop recorder.

Comorbidities, including CKD, were identified based on clinical documentation in the medical record and were not systematically adjudicated using laboratory or imaging criteria.

### Primary endpoint (randomized comparison)

**Figure 2:**
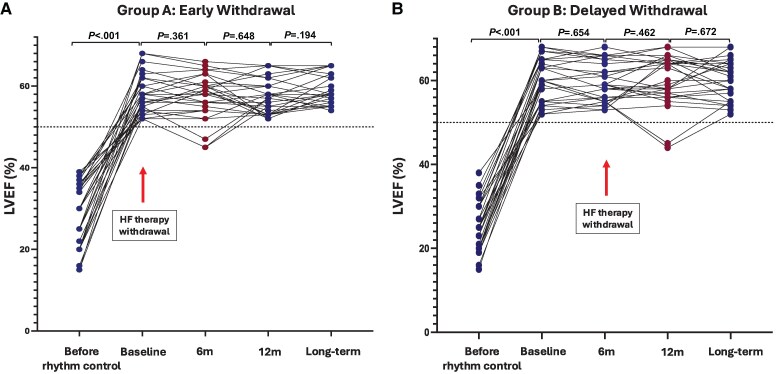
Comparison of left ventricular ejection fraction (LVEF) according to randomization and across study time points. Before rhythm control refers to LVEF at left ventricular systolic dysfunction (LVSD) diagnosis. Timing of catheter ablation was not mandated by the study protocol and varied across participants. Study enrolment occurred following sustained sinus rhythm and LVEF normalization, and subsequent time points reflect standardized study assessments. LVEF was then assessed across study time points and included: enrolment (termed ‘baseline’), 6 months, 12 months and long-term LVEF according to allocation. (*A*) corresponds to Group A with initial medication withdrawal and (*B*) corresponds to Group B, who underwent medication withdrawal at 6 months. Red symbols correspond to values 6 months post-medication withdrawal

During the initial 6-month randomized phase, 27 of 30 participants in Group A maintained LVEF ≥50% (90%), compared with 30 of 30 (100%) in Group B. The OR for LVEF maintenance in Group A vs Group B was 1.18 (95% CI 0.27–2.82, *P* = .47) (*Table 2*). The results of multi-variable regression adjusting for clinical and imaging covariates are outlined in Supplementary data online, Table  *S2*. CMR LVEF was not significantly different between groups at the end of the randomized phase (Group A 58% [95% CI 54–60] vs Group B 59% [95% CI 55–64], *P* = .236; mixed-effects model *P* = .37) (*Figure 2*; Supplementary data online, Figure  *S3*).

**Table 2:** Logistic regression analysis of primary outcome by randomization

Treatment group: study phase 1	LVEF maintenance (%)	OR (95% CI)	*P*-value
Medication withdrawal	27/30 (90%)	1.18 (0.27–2.82)	.47
Medication continuation	30/30 (100%)	Reference	–

Comparison of the primary outcome (LVEF maintenance ≥50% at 6 months) between randomized groups. Odds ratios (ORs) with 95% confidence intervals (CIs) were estimated using logistic regression. This model includes only group assignment as the covariate, consistent with the randomized design. No further multi-variable adjustment was performed due to the low number of outcome events.

### Crossover and safety outcomes

At 6 months, participants crossed over to the alternate treatment arm. Following medication withdrawal in Group B during the second 6-month period, 28 of 30 participants (93%) maintained LVEF ≥50% following medication withdrawal.

A reduction in LVEF to <50% was observed in five participants following short-term medication withdrawal—three from Group A during the initial randomized phase and two from Group B during the crossover withdrawal phase, detected on CMR at 6 months post-medication withdrawal (median LVEF reduction: −7%). The reduction in LVEF was not apparent on TTE performed 4 weeks after medication withdrawal. LVEF reduction was accompanied by an increase in LV end-diastolic volume and elevated NT-proBNP levels (see Supplementary data online, Table  *S3*), but occurred without clinical HF or MACE. Arrhythmia recurrence occurred in one of these five individuals (mean AF burden 4%) leading to initiation of anti-arrhythmic therapy. Characteristics of individuals who relapsed were similar to those without relapse (see Supplementary data online, Table  *S4*).

All five individuals experienced LVEF re-normalization (median LVEF 54%) following re-initiation of HF therapy. A reduction in LVEF >5% was observed in these same five participants (including one with >10% reduction).

On serial CMR imaging, four of the five individuals who relapsed (80%) developed new LV LGE (mid-wall linear LGE in three of four individuals). Two additional individuals without LVEF decline developed new LV LGE at 12-month follow-up (one with focal sub-epicardial/inferolateral LGE and one with fine septal linear mid-wall LGE) (see Supplementary data online, Table  *S5*). Subgroup analysis demonstrated that enrolment LVEF ≤55% was associated with higher risk of LVEF deterioration post-medication withdrawal (OR 5.67, 95% CI 1.10–10.27, *P* = .04) (see Supplementary data online, Figure  *S4*).

There was no MACE or deaths during the study. Three participants were hospitalized during follow-up: one for pneumonia (Group B, during continued HF therapy phase) and two for arrhythmia recurrence after withdrawal. Among participants in Group A, who restarted HF therapy at 6 months per the study protocol, 77% fully recommenced their baseline regimen (see Supplementary data online, Table  *S6*).

### Arrhythmia recurrence

AF recurred in 26 of 60 participants overall (43%), with similarly low AF burden on and off HF therapy (1.4% vs 0.6%, *P* = .50) (see Supplementary data online, Figure  *S5* and Table  *S7*) Rates of cardioversion (*n* = 6) and repeat ablation (*n* = 7) were similarly low between groups and across treatment periods (see Supplementary data online, Figure  *S6*), with no associated LVEF decline.

### Secondary endpoints

Withdrawal of HF therapy was associated with an increase in resting heart rate, systolic and diastolic blood pressure and peak exercise heart rate (*Table 3*). No differences were observed in NT-proBNP, cardiac imaging (CMR/TTE), NYHA class, symptoms and quality of life scores (*Table 3*; Supplementary data online, Figures  *S7–S8*). LV dimensions and NT-proBNP across study time points according to allocation are presented in Supplementary data online, Figures  *S9–S10*. Change in characteristics from baseline to post-medication withdrawal according to allocation is outlined in Supplementary data online, Table  *S8*. Diastolic function characteristics according to allocation are outlined in Supplementary data online, Table  *S9*.

**Table 3:** Haemodynamic and functional measures and clinical outcomes pre- and post-medication withdrawal

	On HF therapy *N* = 60	Off HF therapy *N* = 60	*P*-value
CMR characteristics			
CMR LVEF, %, median (IQR)	58 [54, 62]	59 [55, 64]	.85
CMR LVEDVi, ml/m^2^, median (IQR)	81 [68, 88]	81 [73, 91]	.58
Native T1 time, ms, median (IQR)	1180 [1159, 1207]	1191 [1170, 1210]	.29
Transthoracic echocardiography characteristics			
LVEDVi, ml/m^2^, median (IQR)	54 [46, 69]	55 [45, 66]	.45
LVEDD, mm, median (IQR)	52 [48, 55]	51 [48, 53]	.29
LVMI, g/m^2^, median (IQR)	69 [63, 73]	65 [52, 76]	.48
LV GLS, %, median (IQR)	−18.9 [−17.0, −20.0]	−18.4 [−17.0, −20.0]	.27
RV diameter, mm, median (IQR)	34 [34, 36]	35 [34, 37]	.27
RVs’, cm/s	13 [12, 14]	14 [12, 14]	.01
LAVI, ml/m^2^, median (IQR)	41 [32, 46]	37 [30, 41]	.01
LAA, cm^2^, median (IQR)	24 [21, 28]	23 [20, 26]	.05
RAA, cm^2^, median (IQR)	18 [16, 21]	17 [14, 20]	.01
Haemodynamic and ECG characteristics			
BMI, kg/m^2^, median (IQR)	30 [27, 33]	30 [26, 33]	.27
Resting heart rate, b.p.m., median (IQR)	69 [62, 77]	78 [67, 84]	.005
Resting systolic BP, mm Hg, median (IQR)	126 [118, 132]	132 [127, 142]	<.001
Resting diastolic BP, mm Hg, median (IQR)	80 [75, 86]	85 [80, 88]	.003
QRS duration, ms, median (IQR)	94 [94, 104]	96 [93, 103]	.99
VO_2_max, ml/kg/min, median (IQR)	22.0 [19.1, 26.0]	21.0 [18.6, 25.8]	.40
6MWD, metres, median (IQR)	456 [411, 508]	469 [430, 498]	.54
Cardiac biomarkers			
NT-proBNP, ng/L, median (IQR)	106 [74, 129]	92 [56, 129]	.15
Clinical outcomes			
MACE (incl mortality), *n* (%)	0 (0)	0 (0)	-
AF recurrence, *n* (%)	17 (28)	19 (32)	.69
Median AF burden overall, %	0.0 [0.0, 0.1]	0.0 [0.0, 0.1]	.70
AF burden in recurrence group, %	1.4 [0.2–3.0]	0.6 [0.1–2.9]	.50
Repeat ablation, *n* (%)	3 (5)	4 (7)	.69
Cardioversion, *n* (%)	2 (3)	4 (7)	.47
Quality of life and heart failure symptoms			
SF-36 PCS score, median (IQR)	52 [45, 56]	52 [46, 56]	.70
SF-36 MCS score, median (IQR)	56 [43, 59]	55 [48, 59]	.06
MLHFQ score, median (IQR)	9 [3, 25]	8 [3, 23]	.24

CMR, cardiac magnetic resonance imaging; LVEF, left ventricular ejection fraction; IQR, inter-quartile range; LVEDVi, left ventricular end-diastolic volume index; LVEDD, left ventricular end-diastolic diameter; LVMI, left ventricular mass index; GLS, global longitudinal strain; RV, right ventricular; LAVI, left atrial volume index; LAA, left atrial area; RAA, right atrial area; BMI, body mass index; BP, blood pressure; ms, milliseconds; 6MWD, 6-min walk distance; NT-proBNP, N-terminal pro b-type natriuretic peptide; MACE, major adverse cardiovascular events; AF, atrial fibrillation; SF-36, 36-item Short Form Health Survey; PCS, physical component summary; MCS, mental component summary; MLHFQ, Minnesota Living with Heart Failure Questionnaire.

Values represent CMR-derived measures of left ventricular structure and function obtained at pre-defined study time points. Comparisons were made within individuals while on vs off heart failure (HF) pharmacotherapy. ‘On therapy’ includes CMR data from Group A at baseline (prior to therapy withdrawal) and Group B at 6 months (before crossover). ‘Off therapy’ includes Group A at 6 months (post-withdrawal) and Group B at 12 months (post-crossover and therapy withdrawal).

### Extended follow-up

At 12 months, 55 of 60 participants had maintained LVEF ≥50% off HF pharmacotherapy. Of these, 50 participants remained off HF therapy in consultation with their treating cardiologist, with five individuals resuming all or partial medications for non-HF indications (e.g. hypertension).

**Figure 3:**
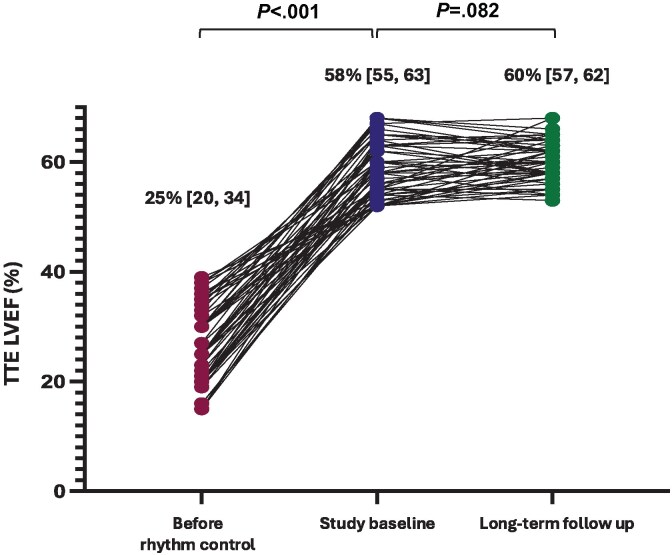
Median left ventricular ejection fraction (LVEF) at left ventricular systolic dysfunction (LVSD) diagnosis, at study enrolment and at long-term follow-up. LVEF before rhythm, at study enrolment and at long-term follow-up in the overall study sample. This combined LVEF measures from Group A and Group B across these time points

During an additional 12-month observational follow-up, all 50 participants remained clinically stable with sustained LVEF normalization (median LVEF 60% [IQR 57–62]) (*Figure 3*; Supplementary data online, Figure  *S11*) and no adverse clinical sequelae.

## Discussion

This is the first prospective randomized study to explore the impact of staged withdrawal of HF therapy in patients with AFCM following LVEF normalization and SR maintenance. The key findings were: (i) in patients with normalized LVEF following AF rhythm control, the majority (90%) exhibited maintenance of LVEF at 6 months in phase 1 following withdrawal of HF therapy; (ii) five patients (three in Group A and two in Group B) exhibited a reduction in LVEF <50% following medication withdrawal across the two study time points, without HF symptoms or adverse clinical sequelae; and (iii) the majority of participants who maintained LVEF and proceeded to the extended follow-up phase continued off HF therapy for an additional 12 months, with stable LVEF observed at 12 months post-study completion (median LVEF 60%) (*Structured Graphical Abstract*).

While guideline-directed HF pharmacotherapy has proven benefits in HF management, it confers potential risks including bradycardia, hypotension, renal impairment, heightened risk of falls, adverse drug reactions or inter-actions, and hospitalization. Additionally, polypharmacy and the associated financial burden place considerable strain on both patients and healthcare systems.^13,14^

Once initiated, HF therapies are typically continued indefinitely, as current guidelines do not specify carefully selected populations in whom medication withdrawal could be considered—a gap that warrants further investigation in future larger studies. This approach may offer several advantages such as reduced side effects, improved adherence to other treatments, a lower financial and pill burden and potential benefits for psychological wellbeing. Importantly, the role of HF pharmacotherapy in patients with AF and LVSD is not unequivocally established, with prior HF pharmacotherapy trials predominantly enrolling patients in SR.^15,16^

A paucity of data exists regarding the effect of withdrawing HF pharmacological therapy in patients with LVSD following LVEF normalization. Earlier non-randomized studies reported deleterious effects of beta-blocker withdrawal in patients with persistent LVSD.^17,18^ Conversely, furosemide withdrawal was not associated with recurrent fluid overload or need for re-initiation in stable mild LVSD and euvolemia.^19^ The REDUCE-AMI study of 5020 patients showed no benefit with long-term beta-blocker use following acute coronary syndrome and LVEF ≥50%^20,21^ and two prior meta-analyses showed no consistent benefit from beta-blocker use on clinical outcomes (mortality and cardiovascular hospitalization) in patients with AF and HF, regardless of LVEF.^22,23^

TRED-HF was the first randomized study of staged withdrawal following LVEF normalization in DCM and reported a 44% relapse rate over 16 weeks.^8^ Of those, very few had AF and the vast majority comprised mixed DCM aetiologies, in which ongoing neurohormonal blockade may have been critical in maintaining LVEF normalization. Conversely, a cohort study of 22 patients with prior peripartum cardiomyopathy and normalized LVEF (>50%) reported no deterioration in LVEF over a median 29-month follow-up post-medication withdrawal.^24^ Similarly, the STOP-CRT randomized pilot study reported LVSD relapse in 7.5% following medication withdrawal at 2 years in patients with prior LVSD following cardiac resynchronization therapy.^25^

The lower relapse rate observed in the present study (8%) compared with TRED-HF (44%) likely reflects fundamental differences in pathophysiology and patient selection. AFCM is characterized by reversible LVSD driven by rate- and rhythm-related mechanisms in the absence of intrinsic myocardial disease. Restoration and maintenance of SR may allow for normalization of cardiac function without the need for indefinite neurohormonal blockade. In contrast, DCM often involves structural myocardial disease, including fibrosis that may persist despite LVEF normalization, placing patients at greater risk of relapse. The rigorous selection criteria applied in this study, incorporating the exclusion of alternative causes of LV dysfunction, absence of myocardial fibrosis on baseline CMR and with a clear temporal relationship between AF onset and LV impairment, likely identified individuals with a high likelihood of AFCM.

Among AFCM, SR restoration promotes LVEF improvement seemingly independent of concurrent HF pharmacotherapy.^3,4,26,27^ Long-term follow-up of the CAMERA-MRI study demonstrated that HF pharmacotherapy alone only modestly improved LVEF at 4 years compared with SR restoration (△LVEF 9% vs 16%; *P* = .008), suggesting its minimal role in LVEF improvement without AF rhythm control.^28^ The present study included a selected population, in whom AF was the primary cause of LVSD, characterized by (i) the absence of CMR-detected myocardial fibrosis; (ii) no alternate cause of LVSD, and (iii) no other indication for HF therapies (such as hypertension or diabetes). These strict inclusion criteria were applied to ensure patient safety and restrict this intervention to patients with a high likelihood of AFCM. However, we acknowledge that the study population was highly selected—all participants underwent CMR to exclude LV myocardial fibrosis, thereby limiting inclusion to those most consistent with an AFCM phenotype. This limits generalizability to real-world populations, where mixed cardiomyopathies are often encountered and AF may coexist with underlying structural heart disease. In such cases, the decision to withdraw HF therapy is more complex and the risk of relapse may be higher. While routine CMR may not always be feasible in clinical practice, it remains a valuable tool for distinguishing reversible AFCM from underlying myocardial pathologies. Broader access to advanced imaging as well as the development of pragmatic clinical or biomarker-driven strategies will be essential to guide individualized treatment decisions in more heterogeneous patient populations with LVEF normalization. In the present study, all patients exhibited LVEF maintenance ≥50% for at least 6 months prior to enrolment. The safety of GDMT withdrawal earlier than or indeed prior to full LVEF normalization post-SR restoration is unclear.

The present study findings contribute to the evolving understanding of rhythm control in patients with varying degrees of systolic dysfunction. In a subgroup analysis stratified by baseline LVEF at the time of LVSD diagnosis, we found that the degree of initial LVEF impairment (<30% vs ≥30%) did not predict the likelihood of relapse following HF therapy withdrawal. Although CASTLE-HTx demonstrated that catheter ablation provides prognostic benefit and improves LVEF in patients with severe LVSD,^29^ these patients had underlying myocardial disease, where AF is a contributor, though not the primary cause of LVSD. In such cases, despite rhythm control, the underlying cardiomyopathy persists, and withdrawal of HF therapy may not be appropriate. In contrast, our study focused on patients with presumed arrhythmia-mediated cardiomyopathy with LVEF following catheter ablation, in whom HF therapy withdrawal may be considered. Extrapolating the present findings to broader HF populations with structural heart disease, where the risk of relapse remains higher, should not be encouraged.

The present study findings contribute to the evolving understanding of rhythm control in patients with varying degrees of systolic dysfunction. In a subgroup analysis stratified by baseline LVEF at the time of LVSD diagnosis, we found that the degree of initial impairment (<30% vs ≥30%) did not predict the likelihood of relapse following HF therapy withdrawal. This is particularly noteworthy in light of recent data from CASTLE-HTx,^29^ which demonstrated that patients with severely impaired LV systolic function and high AF burden derived prognostic benefit from rhythm control with catheter ablation. Importantly, our findings suggest that in the context of successful rhythm control, withdrawal of HF pharmacotherapy may be feasible without significant relapse in select patients. This supports the concept that rhythm control, particularly when AF is the primary driver of cardiomyopathy, may not only facilitate recovery but also allow for de-escalation of therapy in appropriately selected individuals.

These findings should be interpreted within the context of a management strategy that prioritized sustained rhythm control. All patients underwent remote rhythm monitoring using contemporary ECG technology, allowing for early detection of arrhythmia recurrence. In the event of recurrence, rhythm control strategies—including re-ablation, anti-arrhythmic drug therapy, or cardioversion—were promptly initiated and may have mitigated the impact of asymptomatic arrhythmia recurrence.

The potential reversibility of AFCM highlights the importance of early recognition and suggests that timely intervention may improve outcomes. Treatment is centred around early rhythm control and HF pharmacotherapy has historically been implemented as a treatment adjunct, particularly during the recovery phase, partially to restore euvolemia and to support LVEF improvement. Once LVEF normalizes and euvolemia is re-established in this select group, the role of ongoing and indefinite HF therapy is less clear. Moreover, recrudescent LVSD in the absence of arrhythmia recurrence has not been previously examined, particularly in the era of mainstream AF catheter ablation, which is an established effective, safe and guideline recommended treatment for AF with concurrent LVSD.^4,30^ Nearly all patients (97%) underwent catheter ablation as their rhythm control strategy; the remainder demonstrated durable LVEF normalization following electrical cardioversion with anti-arrhythmic therapy. Notably, AF recurrence was relatively common on and off HF therapy (43% overall) though AF burden was low (mean AF burden 1.5%, median 0%) utilizing remote rhythm monitoring. In this setting, early detection and intervention remain critically important and widespread uptake of wearable technologies supports early AF detection and judicious long-term remote rhythm monitoring critical to prevent LVSD relapse in this population.

In the present study, among the five patients who experienced LVSD relapse following short-term medication withdrawal, all were asymptomatic without sequelae and responded to reinstitution of HF pharmacotherapy. In all cases, reintroduction of GDMT, including renin-angiotensin system inhibitors and a beta-blocker, let to prompt improvement in LVEF. Clinical and imaging characteristics, other than LVEF at enrolment, did not differentiate these individuals from the remainder who maintained LVEF following medication withdrawal. Moreover, the primary outcome was unchanged after adjustment for baseline covariates. Adamo *et al*. proposed that LV global longitudinal strain (GLS) was a more sensitive marker of future relapse risk in DCM compared with LVEF alone, albeit this was not an AFCM population^11^ and in the present analysis, as in TRED-HF, LV GLS at enrolment was within the normal range and did not identify the subset who experienced LVSD relapse. However, in the present study, enrolment LVEF was lower in those who subsequently relapsed (LVEF 52% [52, 55] vs 58% [55, 63] among the non-relapse individuals, *P* < .001) and was the only variable that predicted relapse. This may be explained by ultrastructural remodelling and a neurohormonal milieu that may persist within a subset with apparent LVEF normalization, characterized by low-normal LVEF (LVEF 50%–55%) with a likely heightened enduring relapse risk. Moreover, the development of LV LGE on repeat CMR imaging in four of five subjects suggests this minority likely had a concurrent non-ischemic cardiomyopathy rather than purely AFCM.^31^ Moreover, two of the five individuals who experienced relapse in LVSD developed new-onset left bundle branch block, raising the possibility of an underlying pre-disposition to LVSD that initially manifested in the setting of AF and later recurred in association with ventricular dyssynchrony. The emergence of left bundle branch block may have either unmasked a progressive cardiomyopathic process or contributed directly to systolic impairment through ventricular dyssynchrony.

The emergence of recrudescent LVSD without arrhythmia recurrence or baseline LV myocardial fibrosis in this subgroup highlights heterogeneity even among the AFCM population and the inherent challenge of prospectively identifying individuals at risk of relapse despite apparent LVEF normalization. These findings raise the hypothesis that an LVEF threshold >55% could represent a more conservative threshold below which LVEF normalization may be considered incomplete and in whom ongoing HF therapy may be more suitable. Prospective validation of this threshold in larger cohorts is needed. The authors propose that long-term LVEF monitoring (e.g. annually) may be prudent though the optimal surveillance strategy remains to be defined in future prospective studies. Furthermore, genetic evaluation may help identify individuals who might benefit from continued HF pharmacotherapy.

Together, these findings support the feasibility of a cautious, individualized trial of HF therapy withdrawal in carefully selected patients with presumed AFCM. The observation that LVEF decline was reversible with timely reintroduction of therapy provides a practical safety net, offering reassurance to both clinicians and patients that withdrawal can be potentially attempted with appropriate monitoring and prompt re-initiation if needed. These results provide early insights into the potential feasibility of HF therapy withdrawal in AFCM, but they should be viewed as hypothesis-generating. Larger, longer-term studies are needed to identify predictors of sustained recovery, optimal LVEF thresholds for withdrawal and surveillance strategies to ensure safety.

### Limitations

The impact of HF therapy withdrawal on the durability of LVEF normalization beyond 12 months, particularly in the presence of arrhythmia recurrence, remains uncertain. This study did not assess the impact of withdrawing sodium–glucose co-transporter 2 inhibitor therapy given their lack of widespread use as a HF agent at the time this study was performed. The open-label design may have influenced self-reported outcome measures. In light of the large number of secondary outcomes evaluated, we opted not to apply the Bonferroni correction for comparison of individual secondary outcomes to avoid overly conservative thresholds that could increase the Type II error rate. Intermittent remote rhythm monitoring via Kardia transmissions may have underestimated the true AF burden. The modest study sample and 12-month follow-up duration limit the ability to detect infrequent adverse cardiovascular events. The absence of LV LGE on screening CMR does not definitively exclude a possible mixed aetiology for LVSD (such as transient prior myocarditis), which may explain the reduction in LVEF seen in five individuals, four of whom occurred in the absence of arrhythmia recurrence. Larger studies with extended follow-up are warranted to more definitively establish the long-term safety of HF medication withdrawal in this population.

## Conclusion

In this randomized study of patients with rhythm-controlled AF and normalized LVEF following SR restoration, short-term withdrawal of HF therapy was undertaken, with LVEF maintenance in the majority and an LVEF decline in a small minority without clinical sequelae and in the absence of arrhythmia recurrence. These findings suggest that HF therapy withdrawal may be feasible in selected patients with AFCM and normalized LVEF, but further studies are required before clinical recommendations can be made.

## Supplementary Material

ehaf563_Supplementary_Data

## Declarations

### Data Availability

The data utilized in this study may be provided upon reasonable request to the corresponding author.

### Pre-registered Clinical Trial Number

The pre-registered clinical trial number is ACTRN12621000896875.
