# Supplementary material for: Withdrawal of heart failure therapy after atrial fibrillation rhythm control with ejection fraction normalization: the WITHDRAW-AF trial
Source: Eur Heart J. 2025 Aug 12;47(2):250–62. doi: 10.1093/eurheartj/ehaf563 (PMC12777703; doi:10.1093/eurheartj/ehaf563)
Supplement: ehaf563_Supplementary_Data [file ehaf563_supplementary_data.docx]

**Supplementary materials**

**Supplementary figures**

**eFigure 1:** Consort diagram

**eFigure 2:** Study design and medication weaning protocol

**eFigure 3:** Change in LVEF according to randomization

**eFigure 4:** Subgroup analyses of the primary endpoint

**eFigure 5**: AF burden according to randomization

**eFigure 6:** Healthcare utilization on and off HF therapy

**eFigure 7:** Comparison of secondary outcomes on and off HF therapy

**eFigure 8:** Quality of life and HF symptoms on and off HF therapy

**eFigure 9:** LVEDD across study timepoints according to allocation

**eFigure 10:** NT-proBNP across study timepoints according to allocation

**eFigure 11:** Long-term subanalysis of median TTE LVEF off HF pharmacotherapy 12 months post study completion

**Appendix 1:** Medication weaning protocol

**Appendix 2:** Medication reintroduction protocol

**Appendix 3:** AF burden quantification

**Supplementary Tables**

**eTable 1:** Study inclusion and exclusion criteria

**eTable 2:** Results of univariate and multivariable analysis

**eTable 3:** Characteristics of individuals who experienced a relapse in LVSD post medication withdrawal

**eTable 4:** Characteristics of patients with and without LVSD relapse

**eTable 5:** Characteristics of subjects who developed LV LGE on repeat CMR imaging

**eTable 6:** Medication regimen at baseline and following medication reinitiation in the Early Withdrawal Group (Group A)

**eTable 7:** AF recurrence during follow up according to allocation and treatment phase

**eTable 8:** Change in characteristics from baseline to post medication withdrawal according to randomization group

**eFigure 1: Consort Diagram**


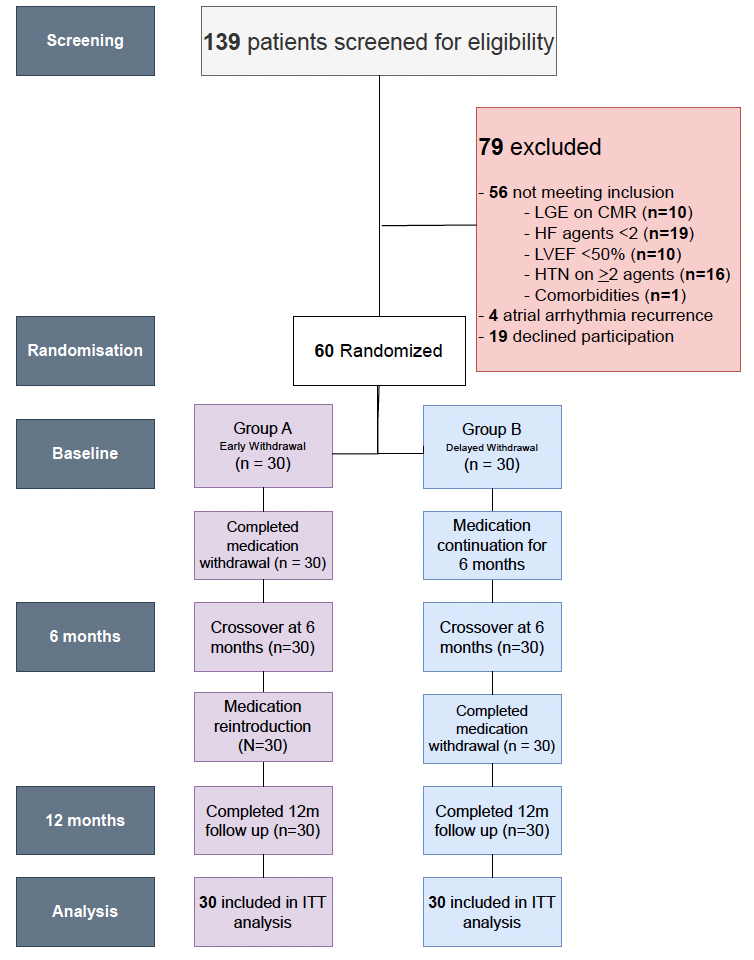


**eFigure 2: Study design and medication weaning protocol**


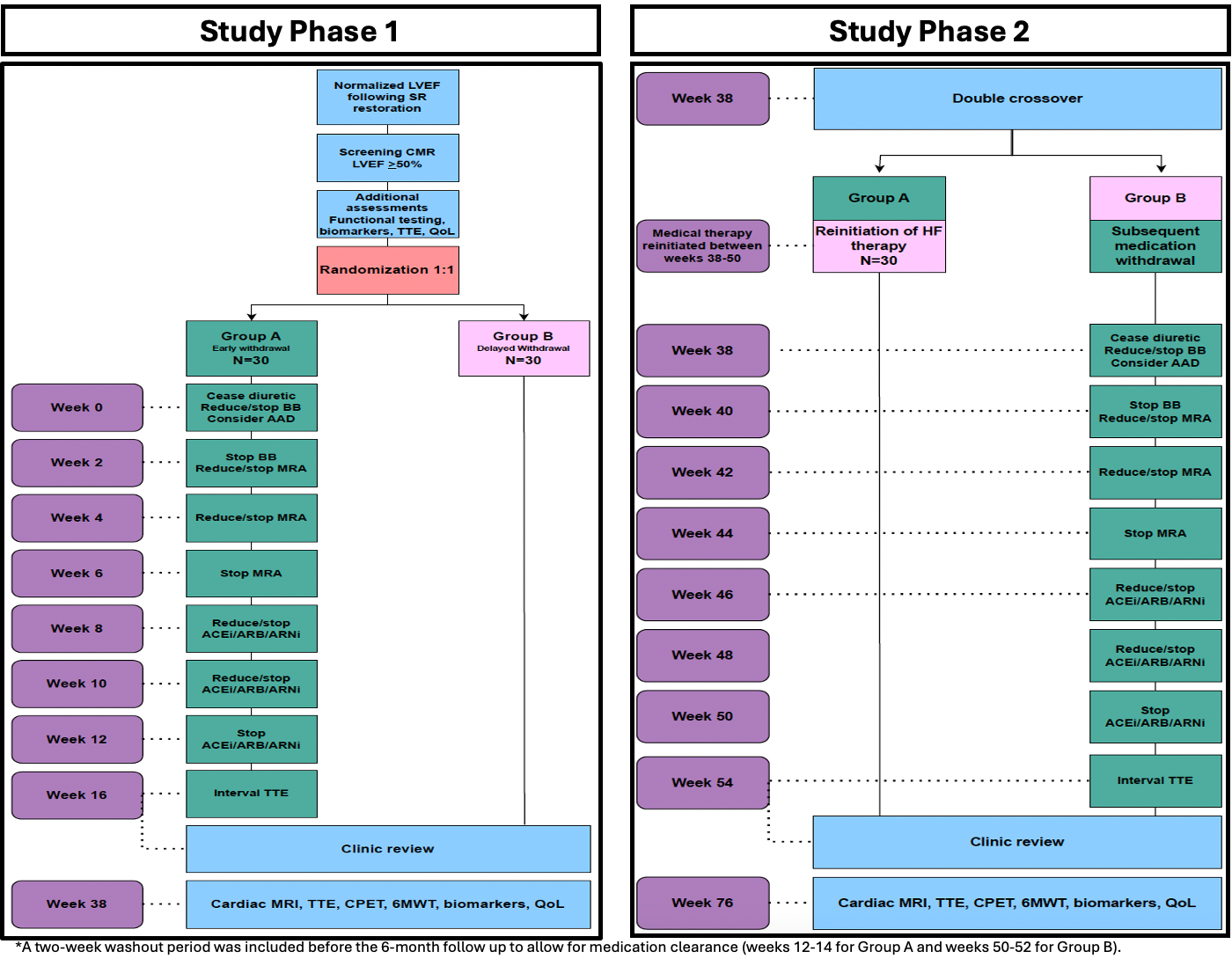


**eFigure 3: Change in LVEF according to randomization**


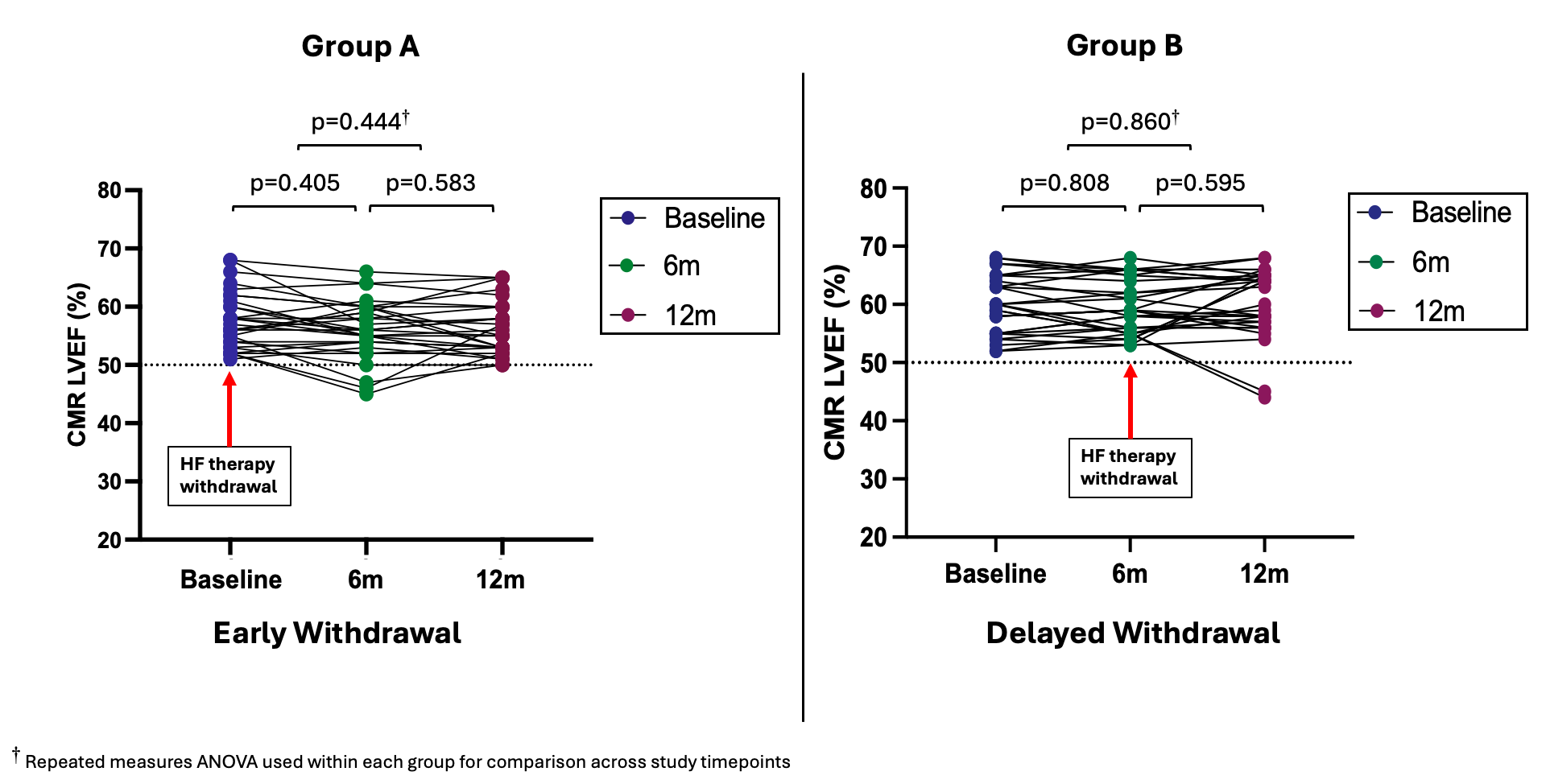


**eFigure 4: subgroup analyses of the primary endpoint**


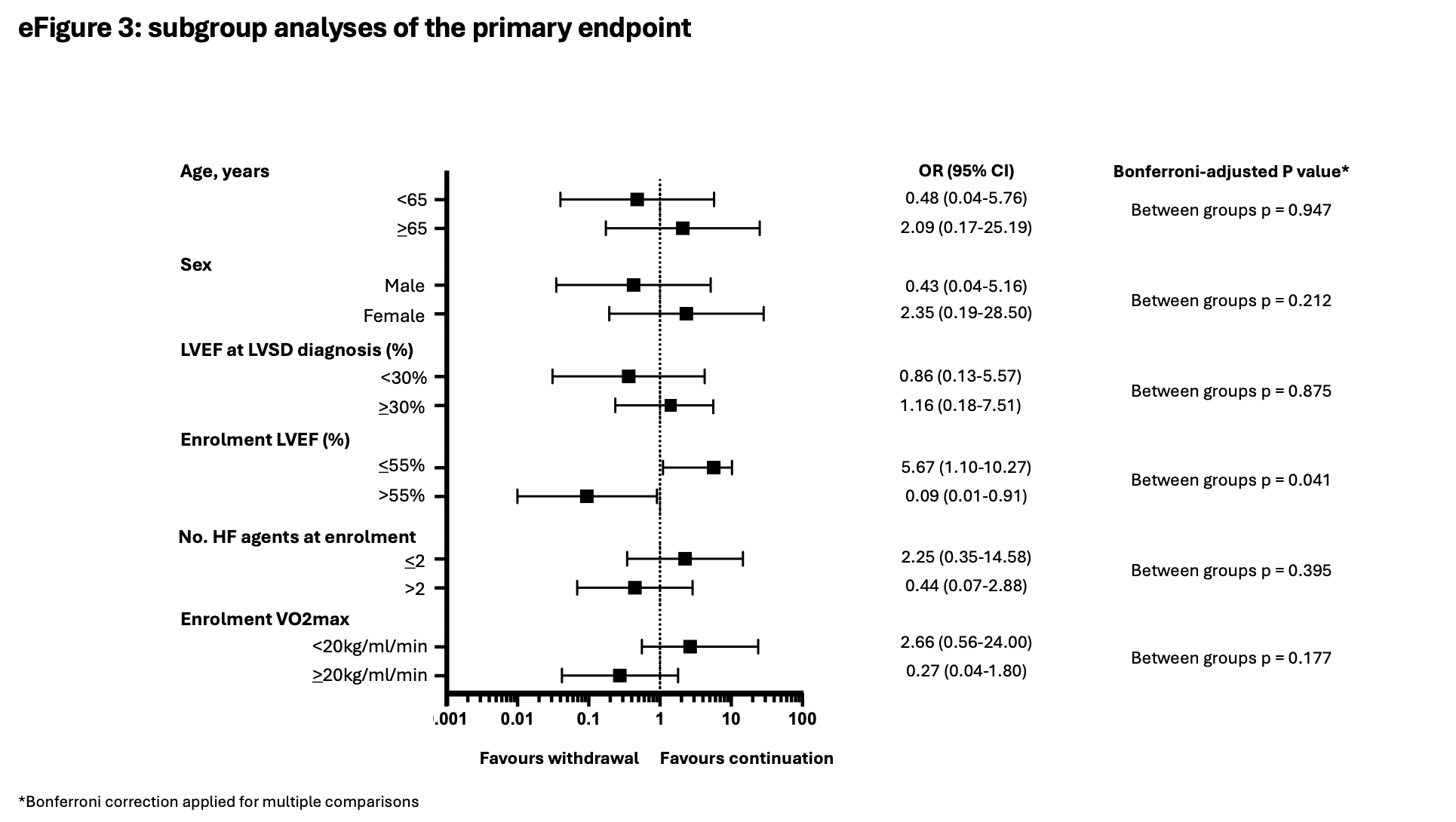


**eFigure 5: AF burden according to randomization**


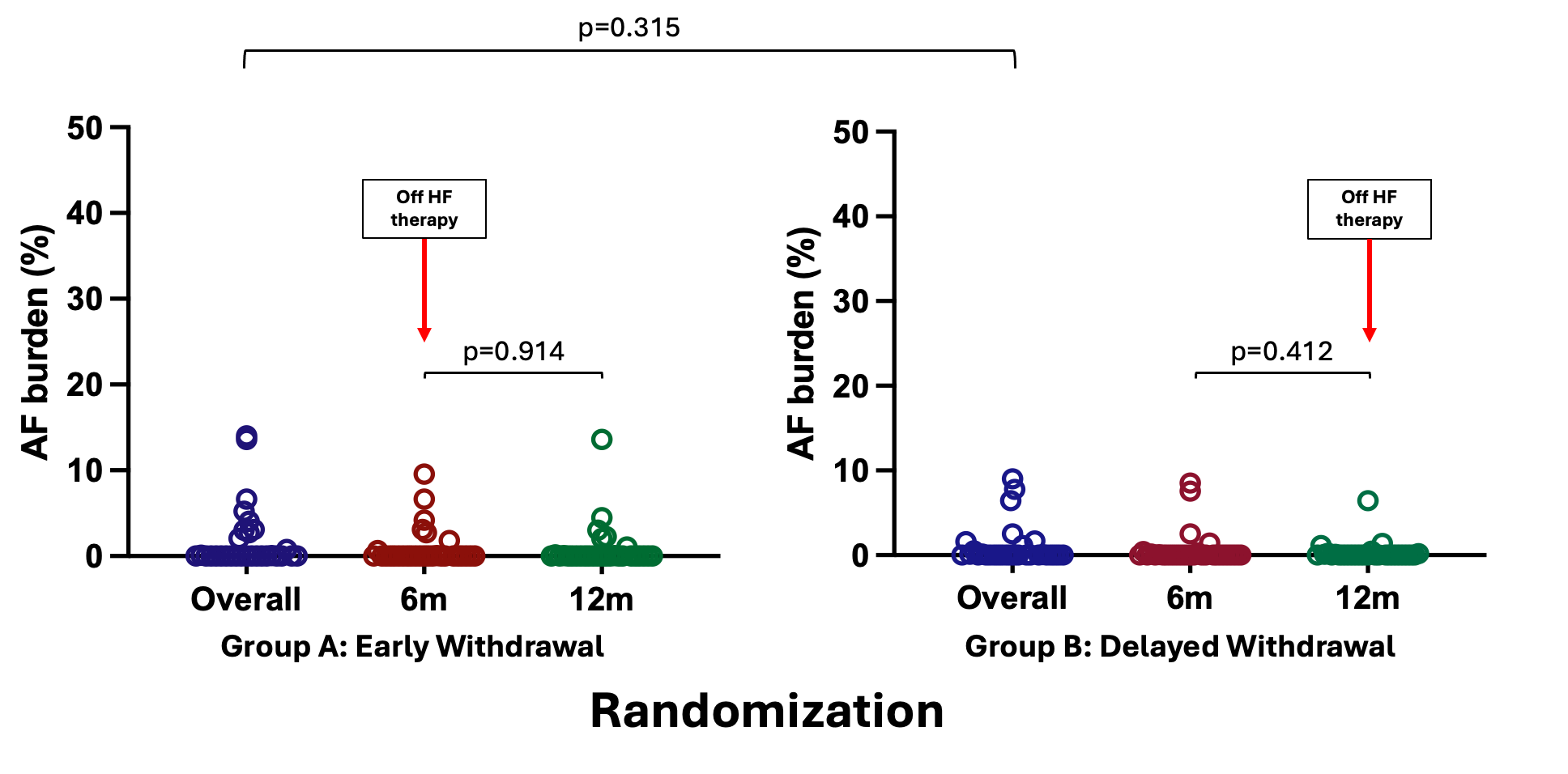


**eFigure 6: Healthcare utilization on and off HF therapy**


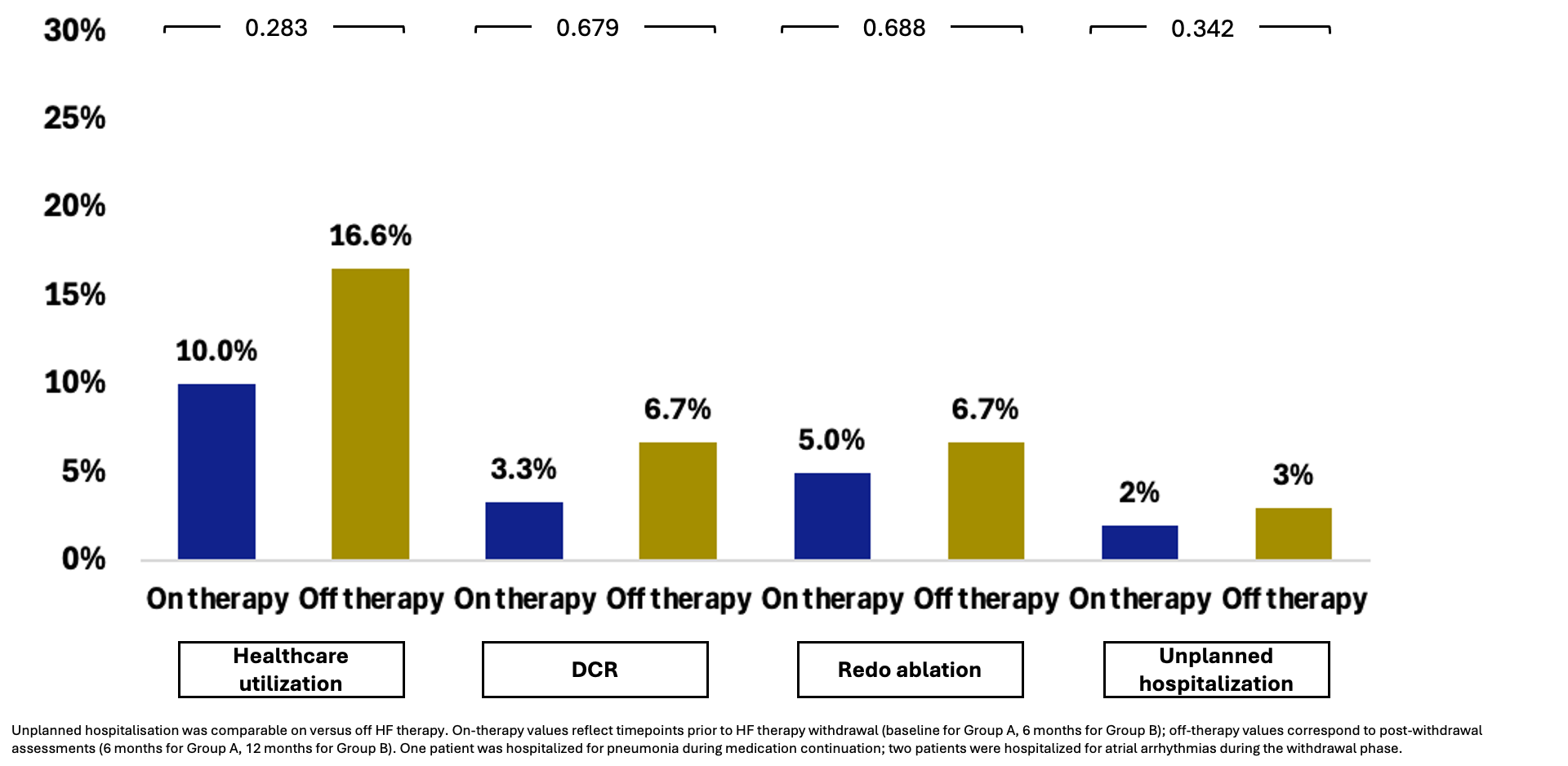


Figure legend: Covariate values are shown for each patient during periods **on** and **off** HF pharmacotherapy using within-subject comparisons to assess characteristics while on versus off HF therapy (each patient served as their own control). For Group A (Early Withdrawal), “on therapy” values reflect at **baseline** and “off therapy” values at **6 months** (after withdrawal). For Group B (Delayed Withdrawal), “on therapy” reflects values at **6 months** (during medication continuation) and “off therapy” reflects values at **12 months** (following therapy withdrawal).

**eFigure 7: Comparison of secondary outcomes on and off HF therapy**


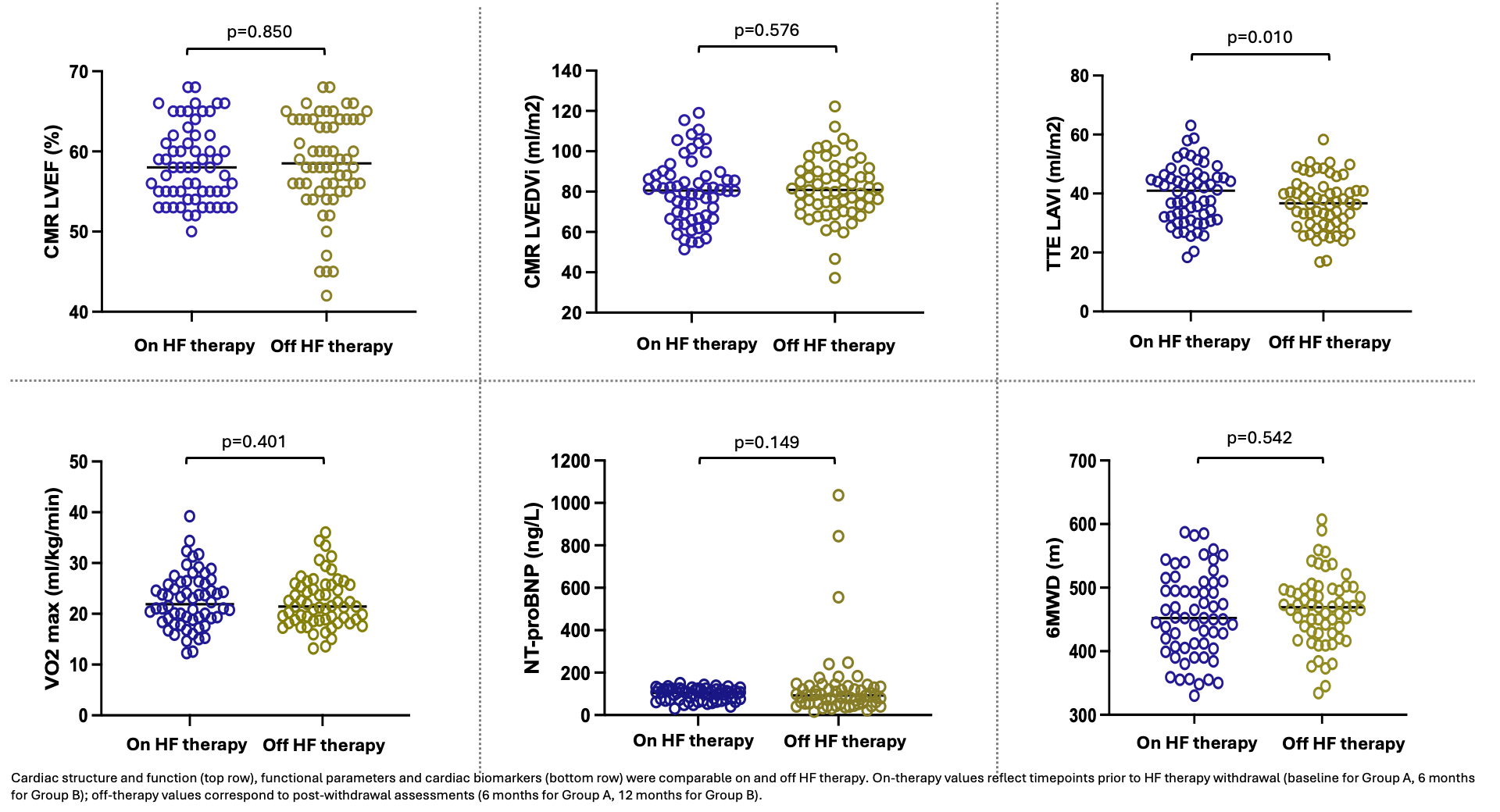


Figure legend: Covariate values are shown for each patient during periods **on** and **off** HF pharmacotherapy using within-subject comparisons to assess characteristics while on versus off HF therapy (each patient served as their own control). For Group A (Early Withdrawal), “on therapy” values reflect at **baseline** and “off therapy” values at **6 months** (after withdrawal). For Group B (Delayed Withdrawal), “on therapy” reflects values at **6 months** (during medication continuation) and “off therapy” reflects values at **12 months** (following therapy withdrawal).

**eFigure 8: Quality of life and HF symptoms on and off HF therapy**


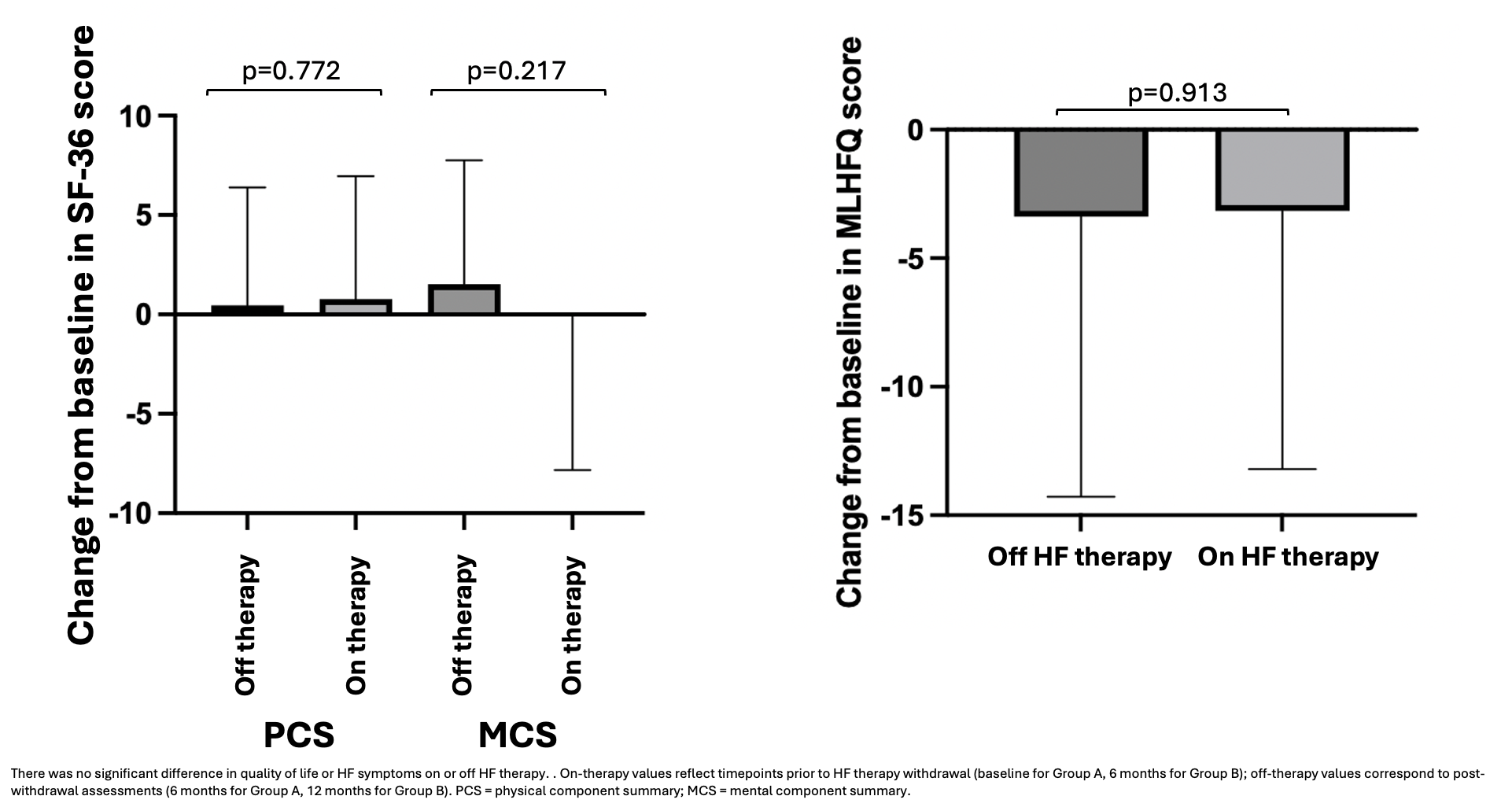


Figure legend: Covariate values are shown for each patient during periods **on** and **off** HF pharmacotherapy using within-subject comparisons to assess characteristics while on versus off HF therapy (each patient served as their own control). For Group A (Early Withdrawal), “on therapy” values reflect at **baseline** and “off therapy” values at **6 months** (after withdrawal). For Group B (Delayed Withdrawal), “on therapy” reflects values at **6 months** (during medication continuation) and “off therapy” reflects values at **12 months** (following therapy withdrawal).

**eFigure 9: LVEDD across study timepoints according to allocation**

**
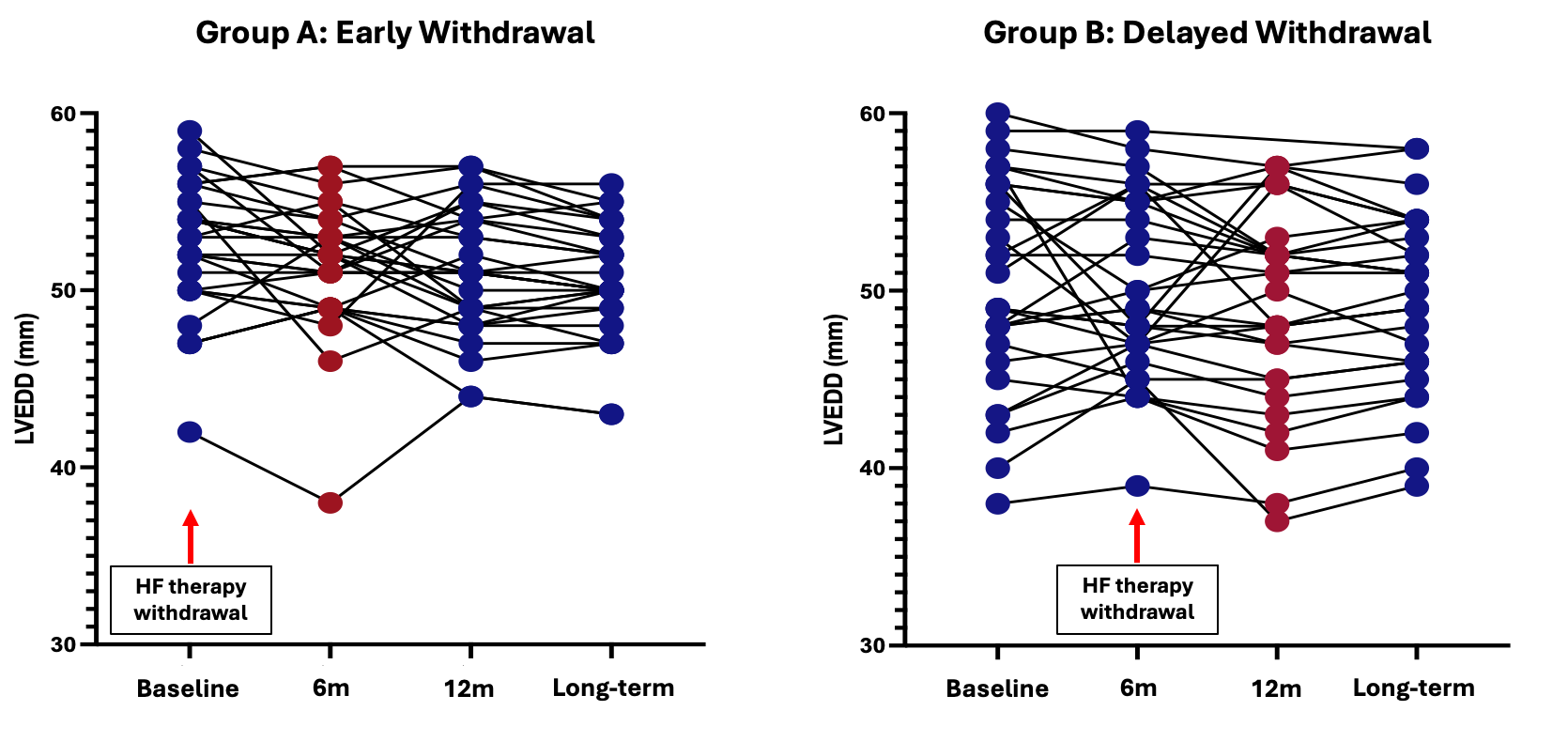
**

**eFigure 10: NT-proBNP across study timepoints according to allocation**

**
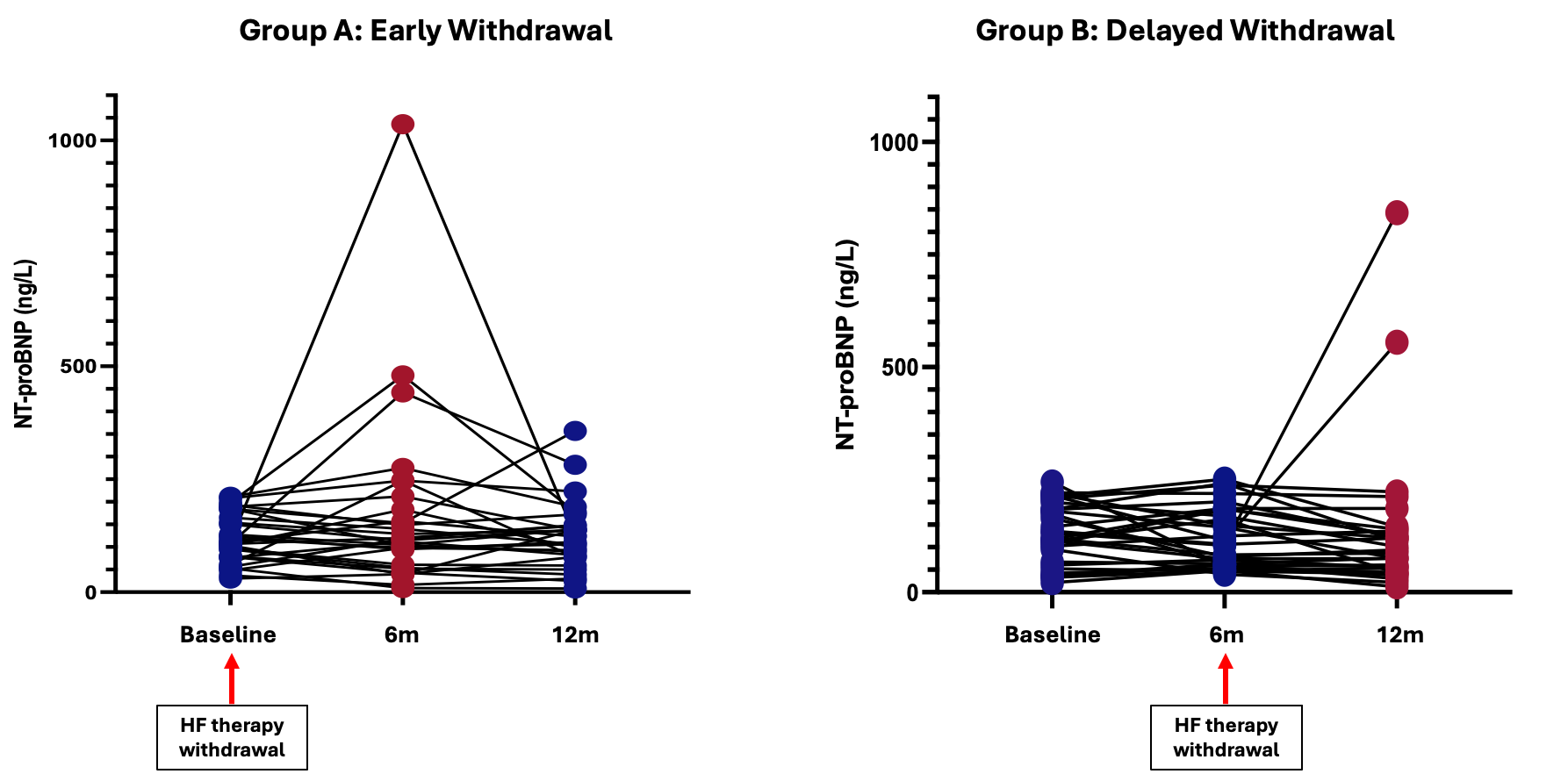
**

**eFigure 11: Long-term subanalysis of median TTE LVEF off HF pharmacotherapy 12 months post study completion**


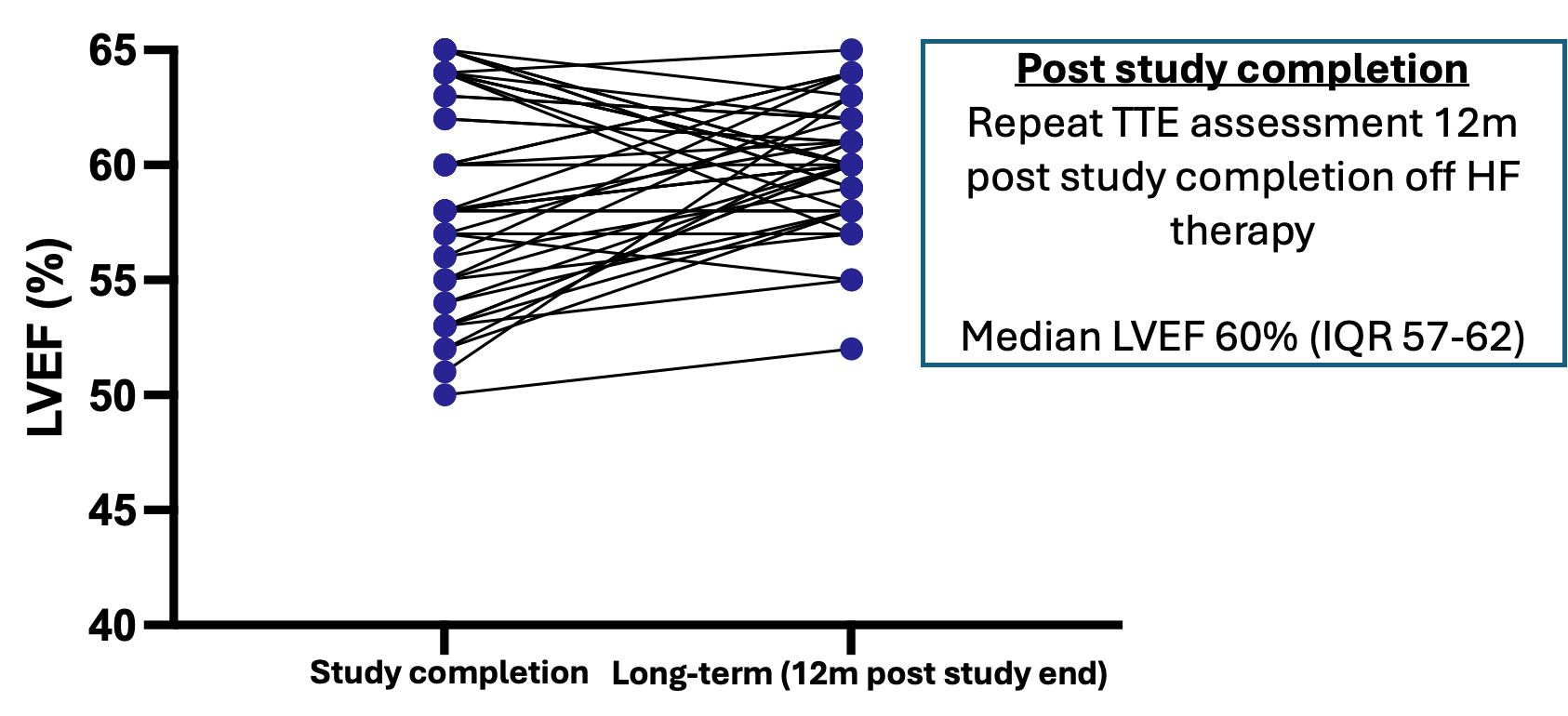

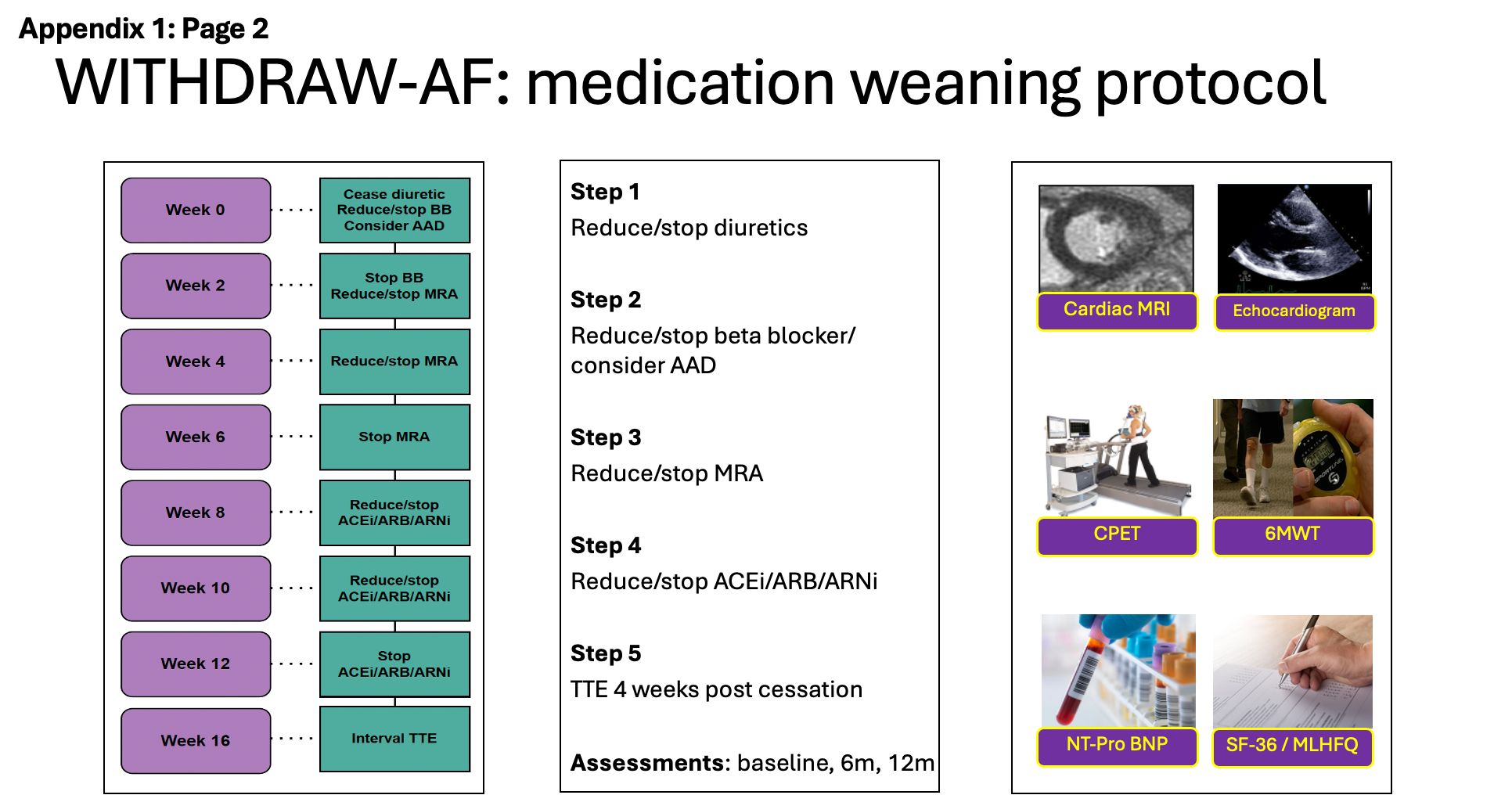

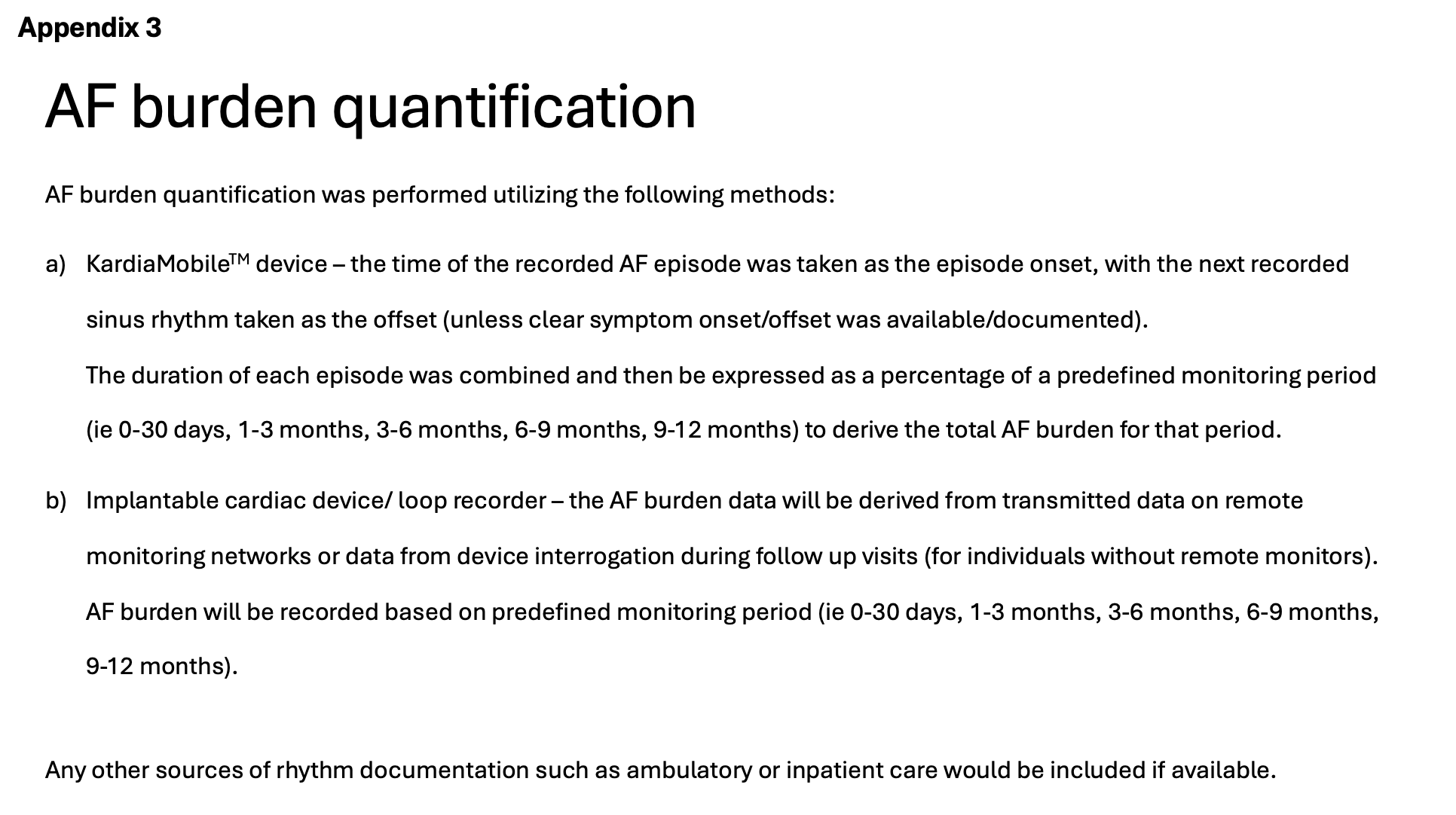


**eTable 1: Study inclusion and exclusion criteria**

| **Inclusion Criteria** | **Exclusion Criteria** |
| --- | --- |
| Age>18 years | Patients unable to maintain sinus rhythm |
| Presumed AF mediated cardiomyopathy -Previous LVEF <40% in the setting of AF  -Recovery to LVEF>50% within 6 months after SR restoration (with anti-arrhythmic medications, electrical cardioversion, catheter ablation or any combination of these) | Patients with known contributing cause of LV dysfunction including   - Ischaemic cardiomyopathy - Valvular heart disease - Hypertrophic cardiomyopathy - Heavy alcohol intake - Other cause of cardiomyopathy (e.g. thyroid disease or myocarditis) |
| NYHA class I | Significant renal impairment (eGFR=30mL/min/1.73m2) |
| Currently on pharmacological anti-heart failure therapy including at least 2 of:   - ACE inhibitor or Angiotensin receptor antagonist - Diuretic (excluding MRA) - Cardiac specific beta blocker - Mineralocorticoid receptor antagonist - Entresto (Sacubitril/Valsartan) | Contraindication to:   - cardiac MRI, - catheter ablation or alternate rhythm control |
| No recurrence of AF with previous 6 months | Any condition with expected survival < 2 years |
| No heart failure related admissions with last 6 months. | Unable to provide informed consent |
| Cardiac MRI demonstrating   - LVEF >50% - The absence of ventricular late gadolinium enhancement - Indexed LVEDV less than 10% upper limit of normal | Patients with a clear indication for ACE/ARB therapy for reasons other than heart failure where alternative agents are contraindicated or inappropriate. |
| Able to consent Willing to adhere to follow up requirements |  |

**eTable 2: Results of univariable and multivariable analysis**

|  | **Univariable analysis** | |  | **Multivariable analysis** | |
| --- | --- | --- | --- | --- | --- |
|  | OR (95% CI) | P value |  | OR (95% CI) | P value |
| Randomization | 1.18 (0.27, 2.82) | 0.471 |  | 1.39 (0.29, 3.45) | 0.642 |
| Age | 1.10 (0.96, 1.26) | 0.185 |  | 1.37 (0.92, 1.41) | 0.123 |
| Female sex | 0.43 (0.04, 5.16) | 0.502 |  | 0.49 (0.02, 4.22) | 0.571 |
| Enrolment LVEF | 1.98 (0.89, 4.43) | 0.196 |  | 1.78 (0.96, 3.31) | 0.131 |
| Enrolment LAVI | 1.05 (0.93, 1.17) | 0.457 |  | 1.10 (0.92, 1.32) | 0.279 |
| Enrolment NT-proBNP | 0.99 (0.98, 1.02) | 0.921 |  | 0.98 (0.95, 1.01) | 0.211 |
| LVEF at LVSD diagnosis | 0.94 (0.79, 1.11) | 0.438 |  | 0.87 (0.63, 1.21) | 0.409 |
| Time to LVEF normalization | 1.50 (0.85, 2.67) | 0.164 |  | 1.69 (0.80, 3.66) | 0.146 |
| Enrolment VO2 | 1.18 (0.90, 1.55) | 0.231 |  | 1.29 (0.80, 2.03) | 0.159 |

**eTable 3: Characteristics of individuals who experienced a relapse in LVSD post medication withdrawal**

| **Endpoint number** | **Baseline** | | | **Endpoint** | | | **Comments** | **LVEF after treatment reinitiation** |
| --- | --- | --- | --- | --- | --- | --- | --- | --- |
|  | **LVEF** | **LVEDVi** | **NT-proBNP** | **LVEF** | **LVEDVi** | **NT-proBNP** |  |  |
| 1 | 52% | 82 | 112 | 45% | 103 | 1036 | New LBBB. No clinical HF. | LVEF 56% |
| 2 | 55% | 77 | 61 | 47% | 82 | 247 | No clinical HF. | LVEF 54% |
| 3 | 52% | 75 | 95 | 45% | 76 | 54 | New LBBB and multifocal PVCs. No clinical HF. | LVEF 50% |
| 4 | 54% | 64 | 115 | 44% | 122 | 555 | No clinical HF. | LVEF 52% |
| 5 | 52% | 69 | 104 | 45% | 84 | 843 | Sinus tachycardia post BB cessation. No clinical HF. | LVEF 55% |

**eTable 4: Characteristics of patients with and without LVSD**

|  | **LVSD Relapse**  **(N=5)** | **No relapse**  **(N=55)** | **P value** |
| --- | --- | --- | --- |
| **Baseline characteristics** | | | |
| Female sex, n (%) | 2 (40) | 9 (16) | 0.19 |
| Age, median (IQR) | 48 [46, 65] | 60 [56, 64] | 0.35 |
| Hypertension, n (%) | 0 (0) | 3 (6) | 0.59 |
| Type 2 Diabetes Mellitus, n (%) | 0 (0) | 3 (6) | 0.59 |
| Hyperlipidaemia, n (%) | 3 (60) | 14 (26) | 0.10 |
| Stroke, n (%) | 0 (0) | 2 (4) | 0.66 |
| OSA, n (%) | 1 (20) | 10 (18) | 0.92 |
| CKD, n (%) | 1 (20) | 2 (4) | 0.11 |
| Alcohol, n (%) | 0 (0) | 7 (13) | 0.40 |
| Smoker, n (%) |  |  | 0.43 |
| Never | 3 (60) | 39 (71) |  |
| Ex-smoker | 2 (40) | 14 (26) |  |
| Current smoker | 0 (0) | 2 (4) |  |
| CHADS2VASc, median (IQR) | 2 [2, 2] | 2 [1, 2] | 0.38 |
| LVEF at LVSD diagnosis, median (IQR) | 25 [23, 35] | 25 [20, 33] | 0.86 |
| Time since LVSD diagnosis, mo, median (IQR) | 21.0 [19.1, 25.3] | 20.0 [14.7, 37.2] | 0.67 |
| Time since LV recovery, mo, median (IQR) | 18.0 [17.3, 18.4] | 14.0 [8.2, 19.8] | 0.77 |
| Enrolment LVEF, median (IQR) | 52 [52, 55] | 58 [55, 63] | <0.001 |
| Enrolment LVEDVi, median (IQR) | 75 [69, 77] | 81 [68, 89] | 0.32 |
| Enrolment LV GLS, median (IQR) | -18.8 [-18.0, -19.6] | -18.4 [-17.0, -20.0] | 0.47 |
| Enrolment LAVI, median (IQR) | 31 [27, 44] | 37 [31, 44] | 0.56 |
| **HF pharmacotherapy** | | | |
| Median no. HF agents, IQR | 3 [2, 3] | 2 [2, 3] | 0.09 |
| 2 agents, n (%) | 2 (40) | 33 (60) |  |
| 3 agents, n (%) | 3 (60) | 14 (26) |  |
| 4 agents, n (%) | 0 (0) | 8 (14) |  |
| **Haemodynamics and cardiac biomarkers** | | | |
| Baseline VO2 max, ml/kg/min, median (IQR) | 18.8 [18.4, 20.0] | 23.5 [19.3, 26.6] | 0.03 |
| Baseline 6MWD, metres, median (IQR) | 384 [374, 450] | 450 [405, 495] | 0.28 |
| Baseline NT-proBNP, ng/L, median (IQR) | 104 [95, 112] | 103 [68, 127] | 0.34 |
| **Arrhythmia recurrence** | | | |
| AF recurrence, n (%) | 1 (20) | 15 (27) | 0.73 |
| Median AF burden overall, % | 0.0 [0.0, 0.0] | 0.0 [0.0, 1.5] | 0.76 |
| **Quality of life and heart failure symptoms** | | | |
| SF-36 PCS score, median (IQR) | 42 [42,47] | 52 [46, 58] | 0.07 |
| SF-36 MCS score, median (IQR) | 50 [40, 54] | 57 [44, 59] | 0.44 |
| MLHFQ score, median (IQR) | 28 [13, 48] | 5 [2, 24] | 0.16 |
| LVEF: left ventricular ejection fraction; LVEDVi: left ventricular end diastolic volume indexed to body surface area; LVEDD: left ventricular end diastolic diameter; LVMI: left ventricular mass index; GLS: global longitudinal strain; RV: right ventricular; LAVI: left atrial volume index; LAA: left atrial area; RAA: right atrial area; BMI: body mass index; BP: blood pressure; 6MWD: 6-minute walk distance; PCS: physical component summary; MCS: mental component summary; MLHFQ: Minnesota Living with Heart Failure Questionnaire. | | | |

**eTable 5: Characteristics of subjects who developed LV LGE on repeat CMR imaging**

| **Case no.** | **Randomization** | **Baseline LVEF** | **Baseline LGE status** | **6m LVEF** | **6m LGE status** | **12m LVEF** | **12m LGE status** | **LGE pattern** |
| --- | --- | --- | --- | --- | --- | --- | --- | --- |
| 1 | Group A* | 52% | No LGE | 45% | No LGE | 55% | LGE present | Minor basal linear midwall LGE |
| 2 | Group A* | 55% | No LGE | 47% | LGE present | 56% | LGE present | Minor subendocardial mid anterior wall |
| 3 | Group A* | 52% | No LGE | 54% | LGE present | 53% | LGE present | Minor septal linear midwall LGE |
| 4 | Group B^ | 55% | No LGE | 55% | No LGE | 44% | LGE present | Midwall basal to mid inferolateral LGE |
| 5 | Group B^ | 52% | No LGE | 55% | No LGE | 45% | LGE present | Minor basal linear midwall LGE |
| 6 | Group B^ | 53% | No LGE | 54% | No LGE | 64% | LGE present | Focal subepicardial inferolateral wall |

*Group A = Early Withdrawal (withdrawal in study phase 1).
^Group B = Delayed Withdrawal (withdrawal at 6m, phase 2).

**eTable 6: Medication regimen at baseline and following medication reinitiation in the Early Withdrawal Group (Group A)**

| **Group A: Early Withdrawal**  **N=30** | | |
| --- | --- | --- |
|  | **Baseline pharmacotherapy** | **Following medication reinitiation** |
| ACEi/ARB/ARNi, n (%) | 30 (100) | 30 (100) |
| <50% target dose | 13 (43) | 14 (47) |
| >50% target dose | 17 (57) | 16 (53) |
| Beta blocker, n (%) | 30 (100) | 29 (97) |
| <50% target dose | 11 (37) | 12 (40) |
| >50% target dose | 19 (63) | 17 (57) |
| MRA, n (%) | 10 (33) | 8 (27) |
| <50% target dose | 3 (30) | 2 (25) |
| >50% target dose | 7 (70) | 6 (75) |
| AAD, n (%) | 3 (10) | 8 (27) |

**eTable 7: AF recurrence during follow up according to allocation and treatment phase**

|  | **Group A**  **Early Withdrawal** | **Group B Delayed Withdrawal** |
| --- | --- | --- |
| **AF recurrence, n (%)** | 13/30 (43%) | 13/30(43%) |
| **AF recurrence (off drug), n (%)** | 9/30 (30%) | 9/30 (30%) |
| **AF recurrence (on drug), n (%)** | 9/30 (30%) | 9/30 (30%) |
| **Proportion with AF recurrence both on and off drug, n (%)** | 5/30 (17%) | 5/30 (17%) |
| **Time to AF (off drug), days, median (IQR)** | 86 [49,135] | 84 [52,111] |
| **Time to AF (on drug), days, median (IQR)** | 46 [18,75] | 51 [26,86] |

**eTable 8: Change in characteristics from baseline to post medication withdrawal according to randomization group**

|  | **Group A**  **(Early Withdrawal)**  **(N=30)** | **Group B**  **(Delayed Withdrawal)**  **(N=30)** | **P value** |
| --- | --- | --- | --- |
| **Change in CMR LVEF, %** | -1 [-4, +1] | 0 [-4, +2] | 0.23 |
| **Change in LVEDVi, ml/m^2^** | 0 [-7, +5] | +2 [-4, +7] | 0.61 |
| **Change in TTE LAVI, ml/m^2^** | 0 [-3, +7] | -1 [-6, +6] | 0.58 |
| **Change in VO2 max** | -0.9 [-2.3, 0.6] | -0.5 [-1.7, 0.7] | 0.12 |
| **Change in 6MWD, metres** | +28 [-18, 65] | +2 [-19, 22] | 0.06 |
| **Change in NT-proBNP, ng/L** | -25 [-63, 39] | -18 [-66, 5] | 0.16 |
| **Change in BMI, kg/m2** | -0.3 [-1.3, 0.7] | --0.3 [-2.6, 0.6] | 0.64 |
| **Change in SF-36 PCS** | 0 [-3, +4] | 0 [-3, +1] | 0.29 |
| **Change in SF-36 MCS** | 0 [-2, +3] | 0 [-2, +4] | 0.55 |
| **Change in MLHFQ score** | -2 [-8, 4] | -1 [-6, 1] | 0.43 |
| SF-36 score - higher score represents better quality of life (mental and physical wellbeing).  MLHFQ score – lower score represents fewer HF symptoms  All data above are presented as median (IQR) as appropriate.  Values represent within-group changes in characteristics from **baseline (prior to withdrawal)** to the **post-withdrawal timepoint**, according to randomization. For **Group A** (initial withdrawal), comparisons reflect change from **baseline to 6 months** (after HF therapy withdrawal). For **Group B** (subsequent withdrawal), comparisons reflect change from **6 months to 12 months** (after HF therapy withdrawal). This table summarizes the effect of HF therapy withdrawal on key clinical and biochemical measures within each randomized group. | | | |

**eTable 9: Diastolic characteristics according to allocation at study timepoints**

|  | **Group A**  **Early Withdrawal** | **Group B**  **Delayed Withdrawal** |
| --- | --- | --- |
| **Baseline** |  |  |
| E/e’ | 8 [7, 10] | 8 [7, 9] |
| LAVI | 36 [30, 45] | 38 [32, 42] |
| TR velocity | 2.5 [2.2, 2.6] | 2.4 [2.2, 2.6] |
| MV deceleration time | 206 [185, 226] | 210 [193, 244] |
| Proportion meeting criteria for diastolic dysfunction | 5/30 (16.7%) | 5/30 (16.7%) |
|  | | |
| **6m** |  |  |
| E/e’ | 8 [7, 9] | 9 [8, 10] |
| LAVI | 33 [28, 43] | 37 [30, 48] |
| TR velocity | 2.4 [2.3, 2.7] | 2.4 [2.2, 2.6] |
| MV deceleration time | 212 [191, 226] | 222 [204, 245] |
| Proportion meeting criteria for diastolic dysfunction | 2/30 (6.7%) | 4/30 (13.3%) |
|  | | |
| **12m** |  |  |
| E/e’ | 8 [7, 10] | 9 [7, 10] |
| LAVI | 31 [27, 40] | 35 [31, 45] |
| TR velocity | 2.5 [2.2, 2.6] | 2.5 [2.1, 2.6] |
| MV deceleration time | 210 [179, 238] | 201 [182, 233] |
| Proportion meeting criteria for diastolic dysfunction | 2/30 (6.7%) | 3/30 (10%) |
